# hTERT Peptide Fragment GV1001 Prevents the Development of *Porphyromonas gingivalis*-Induced Periodontal Disease and Systemic Disorders in *ApoE*-Deficient Mice

**DOI:** 10.3390/ijms25116126

**Published:** 2024-06-01

**Authors:** Wei Chen, Sharon Y. Kim, Alicia Lee, Yun-Jeong Kim, Chungyu Chang, Hung Ton-That, Reuben Kim, Sangjae Kim, No-Hee Park

**Affiliations:** 1 The Shapiro Family Laboratory of Viral Oncology and Aging Research, UCLA School of Dentistry, 714 Tiverton Ave., Los Angeles, CA 90095, USA; chenwei304@ucla.edu (W.C.); skim@dentistry.ucla.edu (S.Y.K.); alicialee15@g.ucla.edu (A.L.); yjbest01@snu.ac.kr (Y.-J.K.); rkim@dentistry.ucla.edu (R.K.); 2 Section of Oral Biology, UCLA School of Dentistry, 714 Tiverton Avenue, Los Angeles, CA 90095, USA; jchang@dentistry.ucla.edu (C.C.); htonthat@dentistry.ucla.edu (H.T.-T.); 3 UCLA Jonsson Comprehensive Cancer Center, 10833 Le Conte Ave., Los Angeles, CA 90095, USA; 4 Teloid Inc., 920 Westholme Avenue, Los Angeles, CA 90024, USA; chiron99@hanmail.net; 5 Department of Medicine, David Geffen School of Medicine at UCLA, 10833 Le Conte Ave., Los Angeles, CA 90095, USA

**Keywords:** GV1001, *Porphyromonas gingivalis*, periodontitis, atherosclerosis, Alzheimer disease

## Abstract

GV1001, an anticancer vaccine, exhibits other biological functions, including anti-inflammatory and antioxidant activity. It also suppresses the development of ligature-induced periodontitis in mice. *Porphyromonas gingivalis* (*Pg*), a major human oral bacterium implicated in the development of periodontitis, is associated with various systemic disorders, such as atherosclerosis and Alzheimer’s disease (AD). This study aimed to explore the protective effects of GV1001 against *Pg*-induced periodontal disease, atherosclerosis, and AD-like conditions in *Apolipoprotein* (*ApoE*)-deficient mice. GV1001 effectively mitigated the development of *Pg*-induced periodontal disease, atherosclerosis, and AD-like conditions by counteracting *Pg*-induced local and systemic inflammation, partly by inhibiting the accumulation of *Pg* DNA aggregates, *Pg* lipopolysaccharides (LPS), and gingipains in the gingival tissue, arterial wall, and brain. GV1001 attenuated the development of atherosclerosis by inhibiting vascular inflammation, lipid deposition in the arterial wall, endothelial to mesenchymal cell transition (EndMT), the expression of Cluster of Differentiation 47 (CD47) from arterial smooth muscle cells, and the formation of foam cells in mice with *Pg*-induced periodontal disease. GV1001 also suppressed the accumulation of AD biomarkers in the brains of mice with periodontal disease. Overall, these findings suggest that GV1001 holds promise as a preventive agent in the development of atherosclerosis and AD-like conditions associated with periodontal disease.

## 1. Introduction

GV1001 is a 16-amino acid peptide derived from the catalytic site of human telomerase reverse transcriptase (hTERT) and originally designed as an anti-cancer vaccine to enhance immune responses [1,2,3]. However, it has been found to possess various other biological activities, including anti-inflammatory, anti-apoptotic, antioxidant, and anti-viral properties, and anti-atherosclerotic activity [4,5,6]. Its anti-inflammatory effects have also been demonstrated in different contexts, such as protecting against renal and myocardial ischemia–reperfusion injury [7,8]. GV1001 has also been shown to reduce proinflammatory cytokines in blood lymphocytes and protect neurons from reactive oxygen species (ROS) [6,9]. Additionally, it downregulates enolase1-produced proinflammatory cytokines [10]. In our previous study, we showed that GV1001 prevented the development of ligature-induced periodontitis and atherosclerosis [11]. Recently, it was reported that GV1001 suppressed neuroinflammation, reduced amyloid-β (Aβ) oligomer levels, and decreased phospho-Tau by activating gonadotropin-releasing hormone receptors (GnRHRs) and their downstream signaling pathways in mice [12]. Importantly, GV1001 has been found to be both effective and safe in patients with moderate-to-severe Alzheimer’s disease (AD) [13].

Periodontitis is a chronic local and systemic inflammatory condition that affects the periodontium, which includes the gingivae, bones, and ligaments supporting the teeth [14,15]. This multifactorial disease is characterized by gingival inflammation and progressive loss of alveolar bone, which ultimately leads to tooth loss. The immune-inflammatory response triggered by periodontitis can cause irreversible damage to the supporting tissues of the teeth and result in the destruction of alveolar bone [16]. Importantly, periodontitis is not only restricted to the oral cavity but has systemic implications as well. It has been linked to several systemic diseases, including rheumatoid arthritis, psoriasis, systemic sclerosis, Alzheimer’s disease, and cardiovascular diseases [17,18,19,20,21]. These associations indicate that the chronic inflammation and immune dysregulation seen in periodontitis can contribute to the development and progression of these systemic conditions. In our previous reports, we demonstrated that severe periodontitis in mice was associated with the development and worsening of atherosclerosis, a condition characterized by the buildup of plaque in the arteries [22,23]. This finding suggests that the chronic inflammation and immune response in periodontitis can contribute to the development of cardiovascular diseases. However, this phenomenon was mitigated by the use of an anti-inflammatory and lipid-lowering agent, indicating the potential of targeting inflammation as a therapeutic strategy for both periodontitis and associated systemic conditions.

In a previous study, we conducted an experiment where we topically applied *Porphyromonas gingivalis* (*Pg*), an anaerobic Gram-negative bacterium and one of the ‘red-complex’ of periodontal pathogens, into the gingival pockets of wild-type (wt) mice. This led to the development of periodontal disease and resulted in both systemic and vascular inflammation [24]. *Pg* releases various bacterial toxins, including lipopolysaccharide (LPS), gingipains, and fimbriae, through local secretion and incorporation into complex outer-membrane vesicles (OMVs) [25,26,27]. These factors contribute to the progressive erosion of the periodontium, resulting in dysbiotic changes such as periodontitis. Additionally, they cause the loss of clinical epithelial attachment, alveolar bone, and the release of proinflammatory mediators [28]. *Pg* LPS induces inflammation by triggering innate immune responses through a distinctive Toll-like receptor interaction [29]. 

Our previous findings demonstrated that the local gingival delivery of *Pg* LPS induced periodontitis and alveolar bone loss in *Apolipoprotein E* (*ApoE)-*deficient mice, accompanied by elevated levels of local and systemic proinflammatory cytokines [22,24,30]. *Pg* gingipains, cysteine endo-proteases, exert virulent effects by degrading the extracellular matrix, cleaving numerous anti-bacterial proteins in saliva, and provoking systemic inflammation [31]. *Pg* produces three distinct types of gingipains: Lysine-gingipain (Kgp), Arginine-gingipain A (RgpA), and Arginine-gingipain B (RgpB) [32]. These gingipains collectively degrade gingival tissues, causing epithelial cell detachment, and also break down other proteins such as the complement system, cytokines, and collagen [33]. Furthermore, gingipains play a vital role in bacterial colonization, inactivating host defenses, and contributing to tissue destruction, thus being indispensable for the survival and pathogenicity of *Pg* [34]. Importantly, *Pg* gingipains were identified in the brains of Alzheimer’s disease (AD) patients, and inhibiting *Pg* gingipains resulted in reduced *Pg* colonization and diminished accumulation of dementia markers in the animal brain [13,35]. 

The current study builds upon our previous findings and other reports on GV1001, proposing the hypothesis that GV1001 prevents the development (or reduces the severity) of *Pg*-induced periodontitis and periodontitis-associated systemic disorders, such as atherosclerosis and AD-like conditions in *Apolipoprotein E* (*ApoE*)-deficient mice. To test this hypothesis, our investigation delved into the effects of GV1001 on the development and severity of *Pg*-induced periodontitis, the development and exacerbation of systemic and vascular inflammation and atherosclerosis associated with *Pg*-induced periodontitis, and the accumulation of AD biomarkers (e.g., Aβ42 and p-Tau) in the brains of mice with *Pg*-induced periodontitis.

These studies aim to provide crucial in vivo and in vitro data supporting prospective human clinical trials evaluating the impact of GV1001 on periodontitis and systemic disorders associated with periodontitis. Our laboratory has made significant strides in testing this hypothesis and presents the following results. Furthermore, this research will offer concrete scientific evidence backing ongoing clinical trials assessing the effect of GV1001 on the progression of human AD. More importantly, future clinical trials determining GV1001’s impact on the initiation and progression of atherosclerosis and AD in patients with periodontitis should be considered. 

## 2. Results

As mentioned in the Introduction, *Pg*, an anaerobic Gram-negative perio-pathogen, is known to play a critical role in the development of periodontitis and is closely associated with many systemic disorders, such as atherosclerosis and AD, in humans [36]. Thus, we induced periodontal disease using topical *Pg* inoculation into gingival pockets of *ApoE*-deficient mice to establish *Pg* DNA aggregates in the gingival pocket (*Pg*-induced periodontal disease) and to investigate the effect of GV1001 on the development and severity of periodontal disease.

### 2.1. Topical Application of Pg into the Gingival Pockets Established the Pg Colony Formation in the Pockets, and Systemic Administration Did Not Alter the Colony Formation

**Figure 1:**
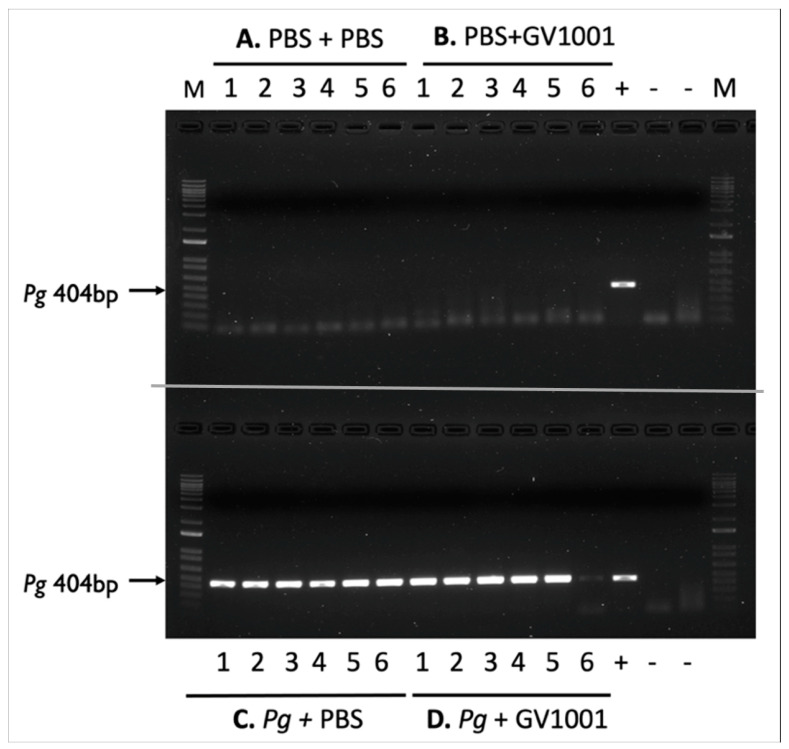
Electrophoretic image of amplified *Pg* DNA from mice sacrificed four weeks after completing the last inoculation (inoculation duration: five weeks). (**A**) PBS + PBS: Swab samples from the mice receiving gingival inoculation with PBS with systemic PBS administration; (**B**) PBS + GV1001: Swab samples from the mice receiving gingival inoculation with PBS with systemic GV1001 administration; (**C**) *Pg* + PBS: Swab samples from the mice receiving gingival inoculation with *Pg* with systemic PBS administration; (**D**) *Pg* + GV1001: Swab samples from the mice receiving gingival inoculation with *Pg* with systemic GV1001 administration. The lanes indicate the following: 1–6, samples taken from the gingival pocket; +, positive control (1000 CFU of purified *Pg* DNA); -, negative control.

To retain the PBS and *Pg* suspension in the gingival pockets following the topical application, we created narrow gingival pockets at the maxillary second molars of mice by placing silk ligatures for one week around the molars. After removal of the silk, we applied PBS or freshly cultured *Pg* suspension topically in the gingival pockets thrice weekly for five weeks. After completing the course of PBS or *Pg* application, we left the mice for four weeks in cages and euthanized them (Supplemental Figure S1). As shown in Figure 1, the swab samples from the mice receiving the topical application of PBS, along with systemic PBS or GV1001 administration for nine weeks, in the gingival pockets did not exhibit any presence of amplified bacterial DNA. All swab samples from the mice receiving *Pg* inoculation exhibited amplified bacterial DNA bands in all tested samples harvested four weeks after the final *Pg* inoculation (six out of six). The amount of detected *Pg*/swab/mouse was ~210,000 CFU. These data indicate that the repeated topical application of *Pg* into the gingival pocket induced and established bacterial colony formation in the mouse gingival pocket. Also, we noted that systemic GV1001 administration did not alter the *Pg* colony formation (detected amount of amplified *Pg* DNA) except for one out of six samples.

### 2.2. Systemic GV1001 Prevented the Increased Periodontal Attachment Loss Induced by Pg Inoculation into the Gingival Pocket

The microscopic findings revealed that the *Pg* inoculation significantly increased periodontal attachment loss (distance from the cementoenamel junction [CEJ] to the base of the gingival pocket) compared to that of the control mice (receiving systemic PBS injection with the PBS inoculation in the gingival pockets). The administration of GV1001 did not alter the periodontal attachment loss in mice compared to that of control mice. However, within the Pg inoculation group, the administration of GV1001 resulted in a statistically significant reduction in periodontal attachment loss (Supplemental Figure S2).

### 2.3. GV1001 Inhibited Pg-Induced Gingival Inflammation by Suppressing the Expression of Proinflammatory Cytokines in the Gingival Tissue

**Figure 2:**
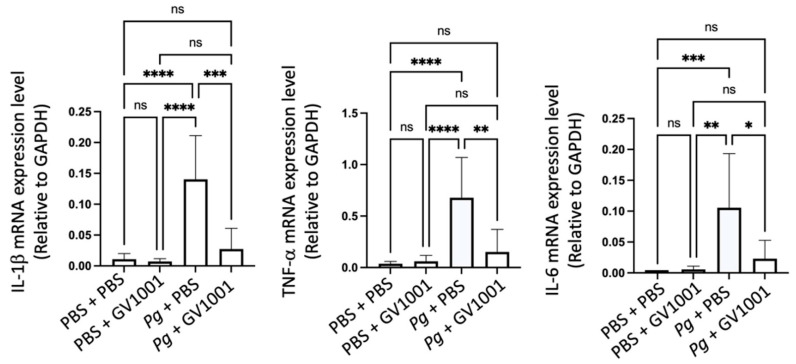
Reversal effect of GV1001 on the enhanced expression of IL-1β, TNF-α, and IL-6 in the gingival tissue induced by *Pg* inoculation into the gingival pocket. * *p* < 0.05, ** *p* < 0.01, *** *p* < 0.001, and **** *p* < 0.0001. ns: not statistically different. Results represent the mean ± SEM performed with *n* = 8–10 mice per group.

To understand the mechanisms of alveolar bone loss by *Pg* and its reversal by GV1001, we investigated the inflammatory status around the *Pg*-inoculated soft tissue by harvesting the gingival tissue around the second molars. Total RNAs were then isolated from the minced gingival tissue and subjected to an RT-qPCR analysis to determine the expression levels of major proinflammatory cytokines (IL-1β, TNF-α, and IL-6). The expression levels of all tested proinflammatory cytokines were notably upregulated by *Pg* inoculation when compared to the control or mice receiving PBS inoculation and systemic GV1001. However, systemic GV1001 administration almost completely abolished the *Pg*-induced increase in the cytokine expression (Figure 2). These data indicate that GV1001 prevents the development of local inflammation induced by topical *Pg* inoculation into the gingival pocket. 

To further validate the above RT-qPCR data, we conducted immunofluorescent staining of IL-1β, TNF-α, and IL-6 from the paraffin sections of maxillary tissue as described in the Materials and Methods section. As shown in Supplemental Figure S3, the topical *Pg* inoculation notably increased the immunostaining of the tested proinflammatory cytokines compared to the control (PBS + PBS) when analyzed with ImageJ software Version 1.52 (National Institutes of Health). As in the data from the RT-qPCR, GV1001 (*Pg* + GV1001 group) remarkably reduced the proinflammatory protein levels compared to the *Pg* + PBS group. GV1001 (PBS + GV1001 group) did not alter the staining compared to the control group. These data indicate that the prevention of *Pg*-induced deep pocket formation and the loss of alveolar bone by GV1001 could be partly due to GV1001’s preventive effect on *Pg*-induced local inflammation. 

### 2.4. GV1001 Reduced the Number of Pg DNA Aggregates and the Aggregates of Pg LPS and Gingipains in the Gingival Tissues

**Figure 3:**
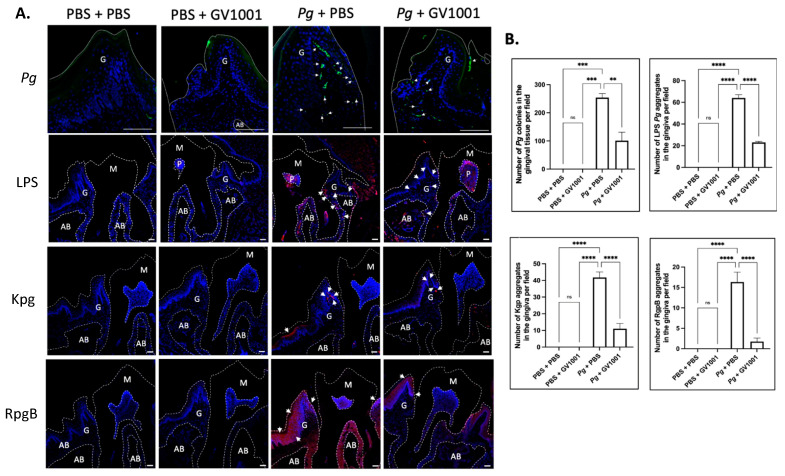
(**A**) Representative FISH staining images of *Pg* colony numbers and immunofluorescent images of LPS, Kgp, and RgpB aggregates in the gingival tissue. Scale bar: 100 μm; M: molar; AB: alveolar bone; G: gingival tissue. Nuclei were counterstained with DAPI (blue dots). Bright green color dots (with white arrows) are *Pg* DNA aggregates. Red colors are aggregates of LPS, Kgp, and RgpB. M: maxillary second molar; G: gingival tissue; P: pulp; AB: alveolar bone. White dashed lines are used to separate different tissues. Bar: 100 μm. (**B**) Results represent the means ± SEM. Statistical analysis was performed with one-way ANOVA from 6 samples of each group. ns: not significantly different; ** *p* < 0.01; *** *p* < 0.001; **** *p* < 0.0001.

To understand whether the preventive effect of GV1001 on *Pg-*induced gingival inflammation is related to the impact of GV1001 on the accumulation of *Pg* DNA aggregates and *Pg*-exotoxins in gingival tissue, we determined the number of *Pg* DNA aggregates and *Pg* LPS and gingipain aggregates in the gingival tissue from different groups. The FISH analysis demonstrated notably high numbers of *Pg* DNA aggregates in the gingival tissue of mice with *Pg* inoculation, which systemic GV1001 significantly reduced. Moreover, immunofluorescence staining showed that Pg LPS and gingipains were abundantly present in the gingival and pulp tissues after Pg inoculation, but were markedly decreased by GV1001 treatment (Figure 3).

### 2.5. Systemic GV1001 Administration Significantly Inhibited the Alveolar Bone Loss Induced by Topical Pg Inoculation into the Gingival Pocket

**Figure 4:**
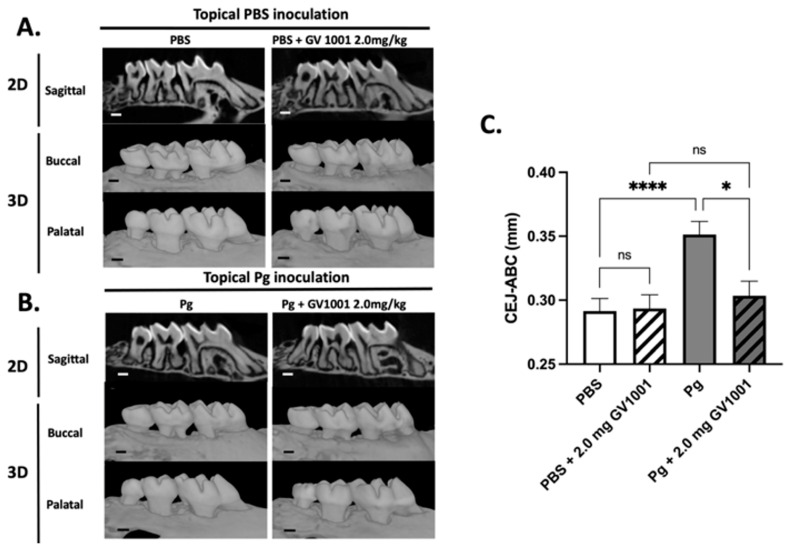
(**A**) Representative two- or three-dimensional *μ*CT images of mice maxillae with PBS inoculation into the pockets and systemic PBS or GV1001 administration. (**B**) Representative two- or three-dimensional μCT images of mice maxillae with *Pg* inoculation into the pockets and systemic PBS or GV1001 administration. (**C**) The average distance (unit: mm) from the palatal and buccal CEJ to the ABC of the second molar. Results represent the means ± SEM performed in ten samples. **** *p* < 0.0001, * *p* < 0.1. ns: not significantly different (*p* > 0.05); CEJ: cement–enamel junction; ABC: alveolar bone crest. Scale bar: 0.2 mm.

The μCT analysis revealed that topical *Pg* inoculation into the gingival pocket with systemic PBS administration induced markedly higher amounts of alveolar bone loss around the maxillary second molars compared to the control mice (PBS inoculation into the pocket and systemic PBS administration). Systemic administration of GV1001 to the mice receiving PBS inoculation did not alter the alveolar bone levels compared to the control, but it completely prevented the bone loss induced by the *Pg* inoculation (Figure 4).

### 2.6. Pg Inoculation Increased the Number of Osteoclasts in the Alveolar Bone, Which GV1001 Significantly Inhibited

To understand the underlying mechanisms of the effect of GV1001 on *Pg*-induced alveolar bone loss, we determined the number of osteoclasts in the alveolar bone of the various groups. As shown in Supplemental Figure S4, the *Pg* inoculation greatly increased the number of osteoclasts in alveolar bone, and the administration of GV1001 significantly reduced the number of osteoclasts, indicating that the reduction of alveolar bone loss by GV1001 may be, in part, due to the diminished number of osteoclasts. 

### 2.7. GV1001 Inhibited Osteoclastogenesis In Vitro

To find whether the in vivo diminution of the osteoclasts by GV1001 was related to the inhibition of the osteoclast formation, we determined the in vitro effect of GV1001 on the osteoclastogenesis of RAW 264.7 cells. We exposed RAW 264.7 cells to GV1001 (0, 1, 5, or 10 ug/mL) with or without recombinant nuclear RANKL, at a concentration of 50 ng/mL for four days followed by 100 ng/mL for one day. The cells were stained for TRAP (tartrate-resistant acid phosphatase) activity to determine the number of osteoclasts.

The GV1001 treatment significantly inhibited multi-nuclear osteoclast formation induced by RANKL (Supplemental Figure S6). These results suggest that the reduced alveolar bone loss by GV1001 is, in part, due to the suppression of osteoclastogenesis.

### 2.8. GV1001 Inhibited Pg-Induced Systemic Inflammation by Suppressing Proinflammatory Cytokine Levels in Serum

**Figure 5:**
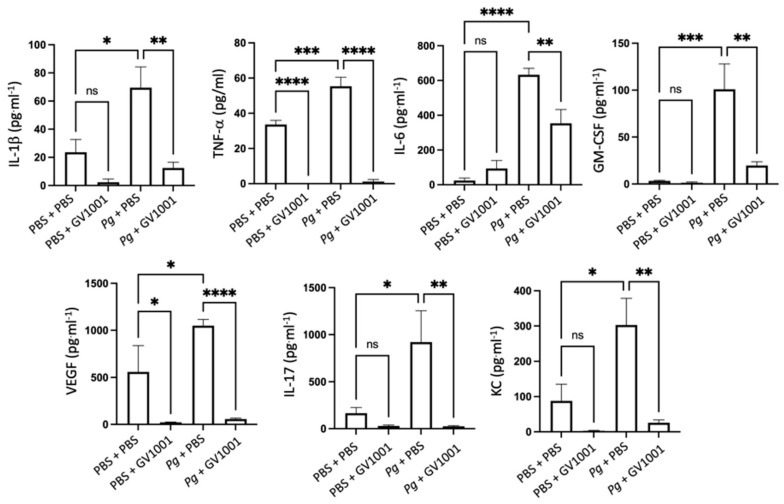
Reversal effect of GV1001 on the increased serum levels of proinflammatory cytokines by *Pg* inoculation into the gingival pockets. Statistical analysis was performed with one-way ANOVA. ns: not significantly different; * *p* < 0.05, ** *p* < 0.01, *** *p* < 0.001, **** *p* < 0.0001.

As periodontitis is both a local and a systemic condition, we determined the effect of *Pg*-inoculation on the induction of systemic inflammation by assessing the levels of proinflammatory cytokines such as IL-1β, TNF-α, IL-6, GM-CSF, VEGF, IL-17, and KC. As expected, *Pg*-inoculation into the gingival pocket induced systemic inflammation by enhancing all tested cytokine levels. Such increases in proinflammatory cytokine levels were almost entirely abolished by systemic GV1001 administration (Figure 5), indicating that GV1001 prevented both the local and systemic inflammation caused by *Pg*-inoculation in the gingival pocket. As we measured the serum cytokine levels four weeks after the final *Pg* inoculation, enhanced systemic proinflammatory cytokine levels in the *Pg* inoculation group may be due to the *Pg* DNA aggregates in the periodontal pockets.

### 2.9. GV1001 Inhibits the Pg-Induced Vascular Inflammation

**Figure 6:**
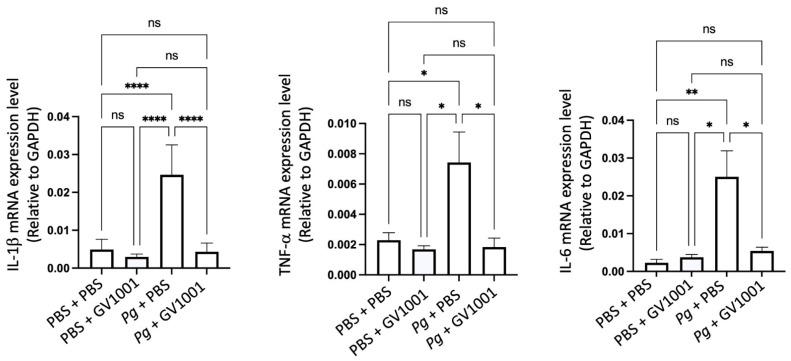
Reversal effect of GV1001 on the increased expression levels of IL-1β, TNF-α, and IL-6 in the arterial wall induced by *Pg* inoculation in gingival pocket. The gene expression was measured with RT-qPCR. GAPDH served as a loading control. Statistical analysis was performed with one-way ANOVA. ns: not significantly different; * *p* < 0.05, ** *p* < 0.01, and **** *p* < 0.0001. Results represent the means ± SEM performed in 5 samples.

Vascular inflammation is known to cause atherosclerosis [37], so we investigated the impact of GV1001 on the development of vascular inflammation by determining the levels of the major proinflammatory cytokines, such as IL-1β, TNF-α, and IL-6. As shown in Figure 6, the *Pg* inoculation greatly increased the expression levels of the proinflammatory cytokines in the arterial tissue. Such an increase was entirely abolished by GV1001, indicating that GV1001 prevented vascular inflammation caused by *Pg*-induced periodontal disease.

**Figure 7:**
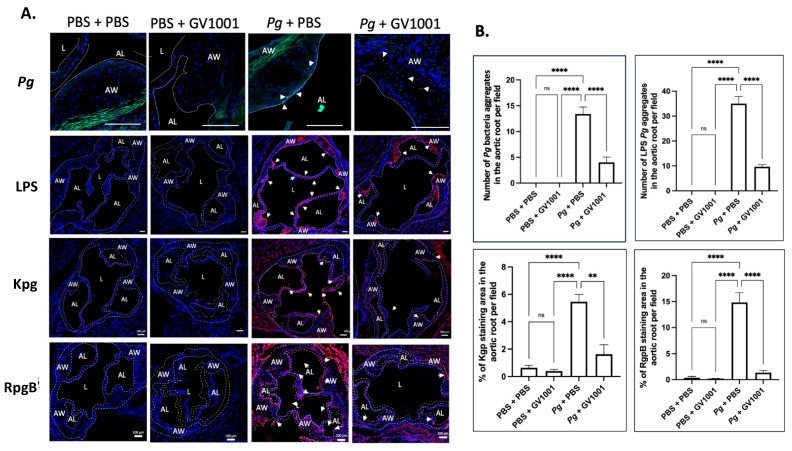
(**A**) Representative fluorescence in situ hybridization (FISH) and immunofluorescent staining images of the *Pg* LPS, Kgp, and RgpB aggregates in the arterial wall images of aortic roots of mice inoculated with *Pg* into the gingival pockets of *ApoE*-deficient mice. Blue dots are nuclei of cells stained with DAPI. *Pg* DNA aggregates are green with white arrows (scale bar: 20 μm), and the *Pg* LPS, Kgp, and RgpB aggregates are bright red color with arrows (scale bar: 100 μm). Nuclei were counterstained with DAPI (blue dots). Green color waves are non-specific staining. AW: arterial wall; L: lumen; AL: arterial leaflet. (**B**) Results represent the means ± SEM of five samples in each group. Statistical analysis was performed with one-way ANOVA. ns: not significantly different; ** *p* < 0.01, **** *p* < 0.0001.

To validate the above RT-qPCR data, we conducted immunofluorescent staining of IL-1β, TNF-α, and IL-6 from the frozen sections of the aortic roots, as described in the Materials and Methods section. As *Pg* can release LPS and gingipains, we first investigated the presence of *Pg* DNA aggregates in the arterial wall from different groups. As shown in Figure 7, we found several *Pg* DNA aggregates in the arterial walls of mice that received *Pg* inoculation, but those colonies were not observed in the mice inoculated with PBS. Systemic GV1001 administration significantly reduced the number of *Pg* DNA aggregates in the arterial wall. *Pg* inoculation also notably increased the intensity of the immunostaining of the tested proinflammatory cytokines when analyzed with ImageJ. As in the data from the RT-qPCR, GV1001 remarkably reduced the proinflammatory protein levels, as shown in Supplemental Figure S5.

### 2.10. GV1001 Reduced the Aggregation Number of Pg DNA, Pg LPS, and Gingipains in the Arterial Wall

To understand whether the preventive effect of GV1001 on *Pg-*induced arterial inflammation is related to the effect of GV1001 on the accumulation of *Pg* DNA aggregates and *Pg*-exotoxins in the arterial wall, we determined the numbers of *Pg* DNA aggregates and aggregates of *Pg* LPS and gingipains in the arterial wall from different groups. The FISH analysis demonstrated that there were many *Pg* DNA aggregates in the arterial wall of mice with *Pg* inoculation, which was notably alleviated by the systemic GV1001 administration. Moreover, the immunofluorescence staining revealed that a high number of *Pg* LPS and gingipain aggregates was detected at the arterial wall of mice with *Pg* inoculation, which GV1001 notably reduced (Figure 7). 

### 2.11. GV1001 Altered the Cholesterol Profiles in Serum: Lowering Total Cholesterol (TC) and Low-Density Lipoprotein (LDL) Levels

Chronic inflammation can contribute to high cholesterol levels by reducing high-density lipoprotein (HDL) while increasing LDL levels. It also triggers the release of substances that accumulate cholesterol in arterial walls, potentially leading to atherosclerosis and heart issues. Although *Pg* inoculation did not significantly enhance the TC and LDL levels compared to the control, we noticed that GV1001 significantly reduced the TC and LDL levels (Supplemental Figure S7). The underlying cause for such alterations remains unknown; we believe that such changes might be related to the anti-inflammatory effect of GV1001. Moreover, such changes caused by GV1001 might affect the lipid deposition (atherosclerosis) in the arterial wall. 

### 2.12. GV1001 Completely Blocked the Pg-Induced Enhanced Lipid Deposition (Atherosclerosis) in the Arterial Wall

**Figure 8:**
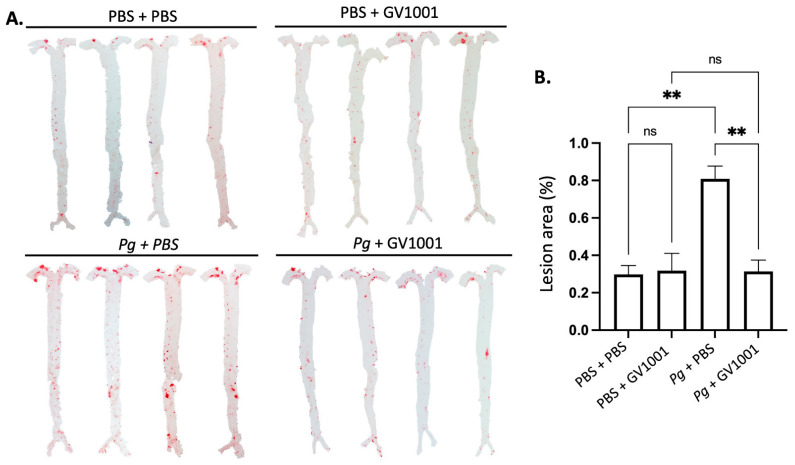
En face analysis of mice arteries. (**A**) Representative photographs of mouse arteries from the en face preparation after staining with Sudan IV (8–10 mice per group). (**B**) Quantification of areas stained by Sudan IV using ImageJ analysis. ns: not significantly different; ** *p* < 0.01.

As vascular inflammation induces atherosclerosis, we investigated the effect of *Pg* inoculation on arterial wall lipid deposition (atherosclerosis) and studied the effect of GV1001 on the degree of lipid deposition in the arterial wall with en face analysis. The full-length of the aorta-to-iliac bifurcation was opened along the ventral midline and dissected free of the animal under a stereomicroscope. Then, the aorta was stained with Sudan IV, as previously described, and pinned out flat, intimal side up, between cover slides [11]. We captured the aortic images with a Nikon digital camera and analyzed them using ImageJ software version 1.48 (NIH, Bethesda, MD, USA; http://imagej.nih.gov/ij (accessed on 19 April 2014)). The percentage of lesion area was calculated as total lesion area divided by total surface area. The en face analysis demonstrated minor lipid deposition in the arterial wall of control mice fed with HFD for ten weeks. Systemic administration of GV1001 (2.0 mg/kg) did not alter the lipid deposition in mice without *Pg* inoculation. The *P*g inoculation induced remarkably higher lipid deposition in the arterial wall compared to the control mice by more than three-fold, and the increase was entirely blocked by 2.0 mg/kg GV1001 administration (Figure 8). 

### 2.13. GV1001 Inhibited the Endothelial–Mesenchymal Transition (EndMT) Induced by TNF-α and Pg LPS

**Figure 9:**
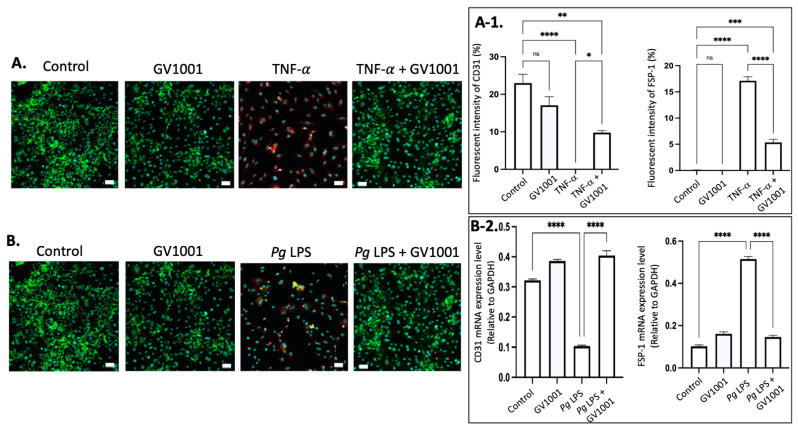
(**A**) Representative immunofluorescent staining images of CD-31 (green), FSP-1 (red), and nucleus (DAPI staining blue) in HUVECs after 48 h exposure of cells to TNF-α with or without GV1001. (**A-1**) Quantification of the fluorescent intensity using ImageJ analysis. (**B**) Representative immunofluorescent staining images of CD-31 (green), FSP-1 (red), and nucleus (DAPI staining blue) in HUVECs exposed to *Pg* LPS for 48 h. (**B-2**) RT-PCR assay was performed for CD31 and FSP-1 mRNA levels in HUVECs exposed to *Pg* LPS for 48h. CD31: endothelial cell marker; FSP-1: mesenchymal cell marker. Scale bar: 50 μm. * *p* < 0.05, ** *p* < 0.01, *** *p* < 0.001 and **** *p* < 0.0001.

It is well known that vascular inflammation induces EndMT [38]. The EndMT will allow the entry of mesenchymal cells, LDL, monocytes, and other leukocytes into the intima of the arterial wall. We previously reported that TNF-α, a major proinflammatory cytokine found in serum and arterial walls, induced the EndMT. Since TNF-α and *Pg* LPS exist abundantly in the arterial wall, we investigated whether TNF-α and *Pg* LPS can induce EndMT in human umbilical venous endothelial cells (HUVECs). As expected, both exposure of cells to TNF-α or *Pg* LPS for 48 h induced EndMT in HUVECs. GV1001 remarkably blocked the in vitro EndMT caused by TNF-α and *Pg* LPS (Figure 9), indicating that the preventive effect of GV1001 on *Pg*-induced atherosclerosis might be partly due to GV1001’s anti-EndMT effect.

### 2.14. GV1001 Inhibited the Uptake of Dil-Ox-LDL by THP-1 Macrophages

**Figure 10:**
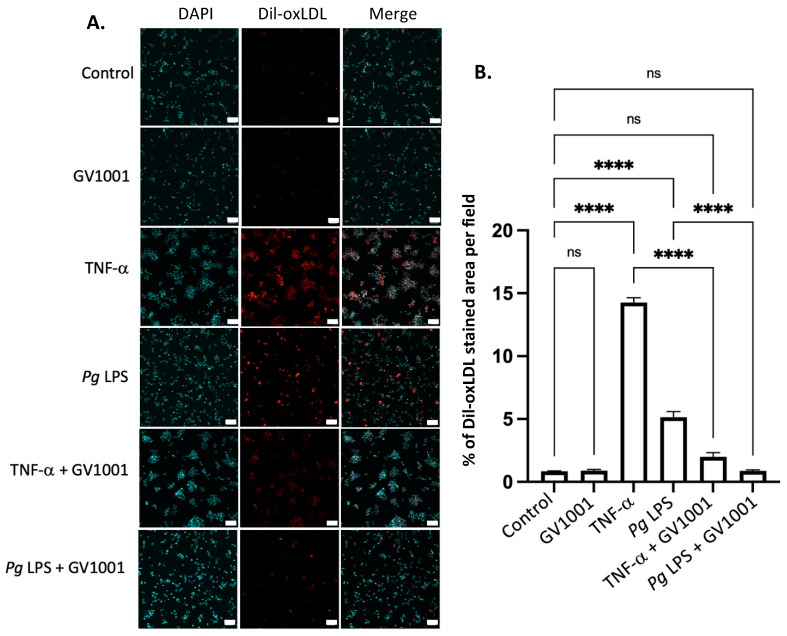
(**A**) Representative immunofluorescent staining images of dil-ox-LDL (Oil Red O staining) in macrophages. A bright red color indicates ox-LDL. Blue dots are nuclei stained with DAPI. (**B**) Quantification of the fluorescent intensity using ImageJ analysis. ns: not significantly different; **** *p* < 0.0001. Scale bar: 60 μm.

As the formation of foam cells from macrophages is a critical step in atherogenesis [39], we investigated the effect of TNF-α, a major proinflammatory cytokine found in the arterial wall, and *Pg*-LPS on the formation of foam cells to understand a mechanism of exacerbation of atherosclerosis in mice with *Pg*-induced periodontal disease. This was determined by assessing the impact of TNF-α and *Pg*-LPS on the uptake of ox-LDL by macrophages differentiated from THP-1 cells. As depicted in Figure 10, TNF-α (100 ng/mL) and *Pg* LPS (10 μg/mL) significantly increased the uptake of ox-LDL by macrophages. GV1001, at a concentration of 10 μg/mL, almost completely inhibited these uptakes, suggesting that GV1001’s inhibition of atherogenesis would be, in part, attributed to its suppressive effect on foam cell formation.

### 2.15. Pg-Induced Periodontal Disease Increased the Level of the Cluster of Differentiation 47 (CD47) in Atherosclerotic Plaques, and GV1001 Reversed the Increase

CD47, an essential anti-phagocytic molecule critical in carcinogenesis, is known to hinder the elimination of cancer cells by macrophages [40,41]. In the advanced stage of atherosclerosis, characterized by the accumulation of arterial smooth muscle cells in the atherosclerotic necrotic core due to impaired clearance [42,43], we assessed the level of CD47 in aortic roots. As illustrated in Supplemental Figure S8, a negligible amount of CD47 was detected in the aortic roots of control mice or mice receiving GV1001 without *Pg* inoculation. However, the immunofluorescent staining level of CD47 in the aortic roots significantly increased in mice with *Pg*-induced periodontal disease compared to the controls, and GV1001 administration remarkably inhibited the staining intensity of CD47 in aortic roots.

**Figure 11:**
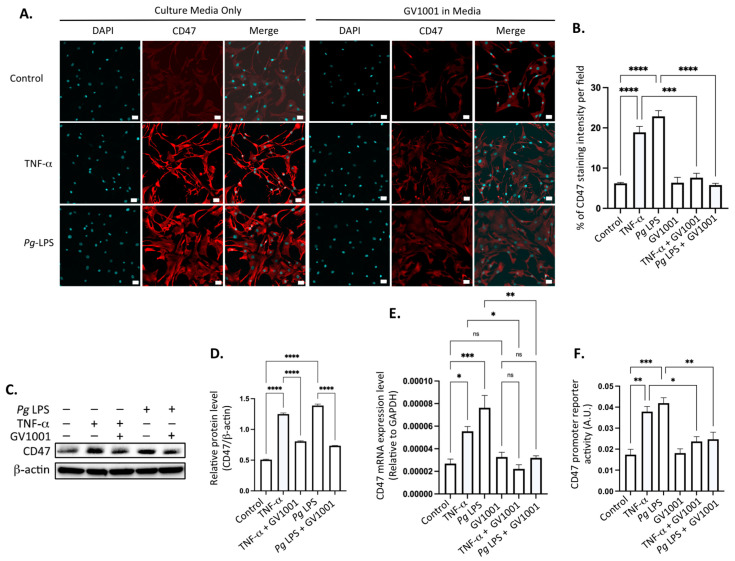
(**A**) Representative immunofluorescent staining images of HCASMCs treated with TNF-α and Pg LPS, both with and without GV1001. Bright red color indicates CD47. Blue dots are nuclei stained with DAPI. (**B**) Statistical analysis of CD47 expression in HASMCs exposed to TNF-α or Pg LPS, comparing the presence and absence of GV1001. (**C**) Western blot analysis illustrating CD47 levels in HCASMCs subjected to TNF-α or Pg LPS, with and without GV1001 treatment. (**D**) Quantification of CD47 protein levels based on Western blot results. (**E**) RT-qPCR analysis evaluating CD47 expression in HCASMCs exposed to TNF-α or Pg LPS, with or without concurrent GV1001 treatment. (**F**) Dual luciferase reporter assays for CD47 promoter activity in HCASMCs treated with TNF-α or Pg LPS, with and without GV1001. ns: not significantly different; * *p* < 0.05, ** *p* < 0.01, *** *p* < 0.001, **** *p* < 0.0001. Scale bar: 50 μm.

To comprehend the mechanisms underlying the inhibitory effect of GV1001 on elevated levels of CD47 in aortic roots in mice with *Pg*-induced periodontal disease, we conducted in vitro studies using human coronary artery smooth muscle cells (HCASMCs). Exposure of these cells to TNF-α (100 ng/mL) or *Pg* LPS (20 μg/mL) significantly increased the CD47 level in HCSMCs. Notably, GV1001 demonstrated complete suppression of CD47 expression in the cells (Figure 11A,B). The suppression was further validated through Western blotting (Figure 11C,D) and RT-qPCR analysis of the CD47 (Figure 11E). Moreover, GV1001 exhibited inhibitory effects on the CD47 gene promoter activity, which had been heightened by TNF-α and *Pg* LPS (Figure 11F). These findings collectively suggest that the downregulation of CD47 expression by GV1001 in arterial smooth muscle cells may contribute, in part, to the prevention of atherosclerosis by GV1001 in vivo.

### 2.16. Pg-Induced Periodontal Disease Induced Neuroinflammation by Increasing the Expression of Proinflammation Cytokines

**Figure 12:**
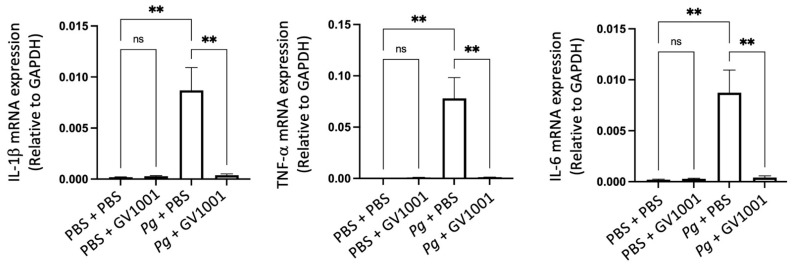
Reversal effect of GV1001 on the enhanced expression levels of IL-1β, TNF-α, and IL-6 by *Pg* inoculation into gingival pocket in whole brain tissues. Statistical analysis was conducted through one-way analysis of variance (ANOVA), where ns indicates not significant, and ** denotes *p* < 0.01. The presented results represented the means ± SEM from 6–10 samples.

Individuals with Alzheimer’s Disease (AD) display neuroinflammation marked by changes in the brain’s proinflammatory cytokine profile [44]. Considering the strong link between Pg, which triggers gingival and systemic inflammation, and human dementia and AD [35,45], our study aimed to investigate whether *Pg*-induced periodontal disease causes neuroinflammation in the mouse brain. The RT-qPCR results indicated a significant increase in the expression of key proinflammatory cytokines, such as IL-1β, TNF-α, and IL-6, in brain tissues as a result of Pg-induced periodontal disease. Notably, GV1001 completely inhibited this increase in the brain tissues of mice with *Pg*-induced periodontal disease (Figure 12). This suggests that GV1001 might have therapeutic potential in reducing the neuroinflammatory response linked to *Pg*-induced periodontal disease in the context of AD.

To validate the RT-qPCR data, we performed immunofluorescent staining for IL-1β, TNF-α, and IL-6 on paraffin sections of brain tissue, as detailed in the Materials and Methods section. Consistent with the RT-qPCR data, *Pg*-induced periodontal disease significantly increased the immunofluorescent staining intensity of these proinflammatory cytokines in the cerebral cortex and hippocampus compared to mice without Pg inoculation. GV1001 markedly reduced the staining intensity of these cytokines in both the cerebral cortex and hippocampus (Supplemental Figures S9 and S10).

### 2.17. Pg-Induced Periodontal Disease Induced an Accumulation of Pg DNA, Pg LPS, and Gingipain Aggregates in the Cerebral Cortex and Hippocampus of Mice, Which Were Notably Reduced by Systemic GV1001

**Figure 13:**
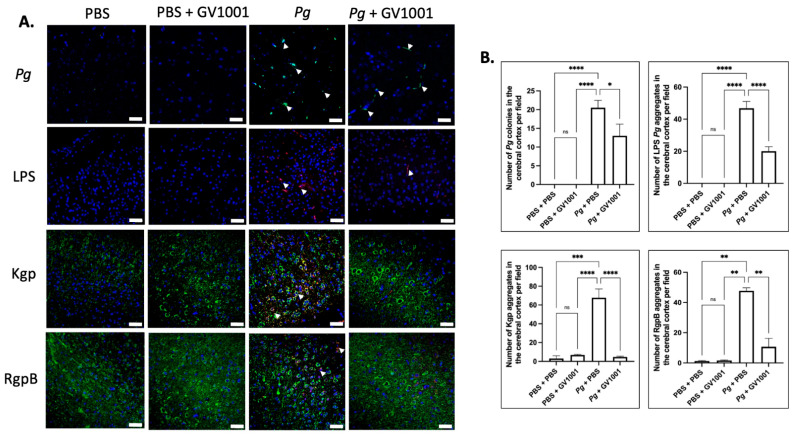
(**A**) Representative FISH images illustrating *Pg* DNA aggregates and immunofluorescent staining images depicting *Pg* LPS, Kgp, and RgpB aggregates in the cerebral cortex of mice with *Pg*-induced periodontal disease. *Pg* DNA aggregates are indicated by the green color with white arrows, while *Pg* LPS, Kgp, and RgpB aggregates are highlighted in bright red with arrows. Nuclei were counterstained with DAPI (blue dots), and neurons are visualized in green. (**B**) Statistical analysis was conducted using one-way ANOVA. Abbreviations: ns, not significantly different; * *p* < 0.05, ** *p* < 0.01, *** *p* < 0.001, **** *p* < 0.0001. Scale bar: 40 μm.

**Figure 14:**
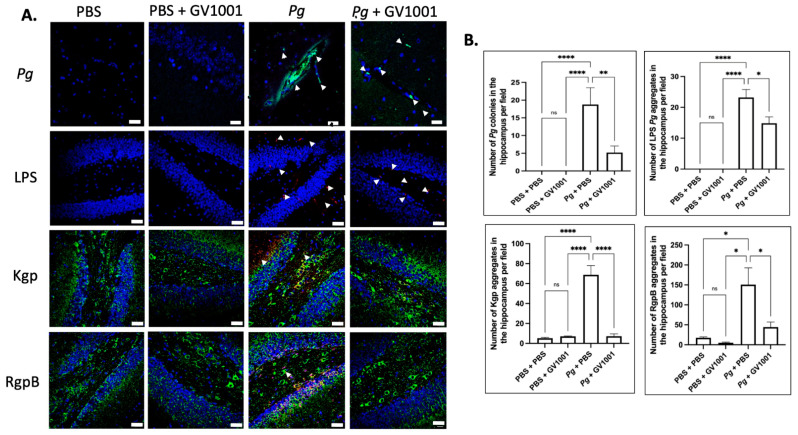
(**A**) Representative FISH images illustrating *Pg* DNA aggregates and immunofluorescent staining images depicting *Pg* LPS, Kgp, and RgpB aggregates in the hippocampus of mice with *Pg*-induced periodontal disease. *Pg* DNA aggregates are shown with the green color with white arrows, while *Pg* LPS, Kgp, and RgpB aggregates are highlighted in bright red with arrows. Nuclei were counterstained with DAPI (blue dots), and neurons are visualized in green. (**B**) Statistical analysis was conducted using one-way ANOVA. Abbreviations: ns, not significantly different; * *p* < 0.05, ** *p* < 0.01, **** *p* < 0.0001. Scale bar: 40 μm.

To examine the potential correlations between the GV1001 mitigating effect on neuroinflammation and the levels of *Pg* DNA aggregates, LPS, and gingipains, we quantified *Pg* DNA aggregates, *Pg* LPS, and gingipains in the brain tissue of mice. As illustrated in Figure 13 and Figure 14, there was a remarkable increase in the aggregate numbers of *Pg* DNA, LPS, and gingipains (Kgp and RgpB) in the cerebral cortex and hippocampus of mice with *Pg*-induced periodontal disease. Notably, systemic administration of GV1001 significantly reduced the numbers of *Pg* DNA aggregates, *Pg* LPS aggregates, and gingipain accumulations in these brain regions.

### 2.18. Pg-Induced Periodontal Disease Induced an Accumulation of Aβ_42_ and p-Tau in the Brain, and GV1001 Notably Decreased the Accumulation

**Figure 15:**
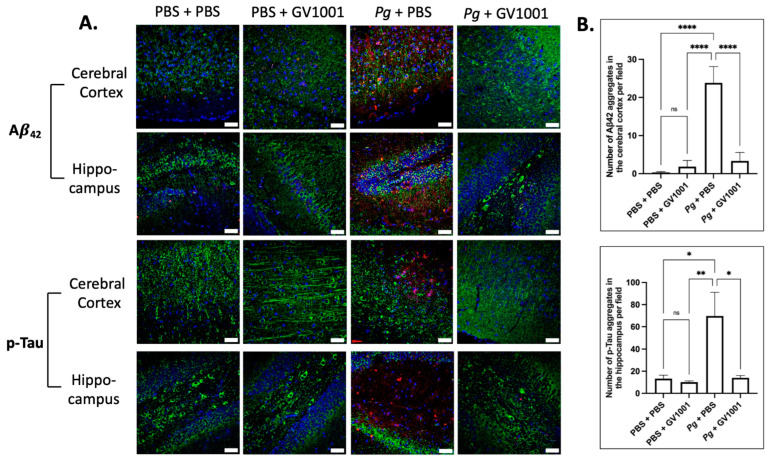
(**A**) Representative immunofluorescence staining images illustrating Aβ42 and p-Tau aggregates in the cerebral cortex and hippocampus of mouse brain. Aβ42 or p-Tau aggregates are depicted as bright red dots, nuclei are stained with DAPI (blue dots), and neurons are marked with MAP2 in green. (**B**) The results are presented as means ± SEM. Statistical analysis was performed using one-way ANOVA based on data obtained from 6 samples in each group. Abbreviations: ns, not significantly different; * *p* < 0.05, ** *p* < 0.01, **** *p* < 0.0001. Scale bar: 40 μm.

Given the presence of neuroinflammation, we anticipated that *Pg*-induced periodontal disease would elevate the levels of Alzheimer’s disease (AD) biomarkers, such as Aβ42 and p-Tau, in the brain. Indeed, the intensity of Aβ42 and p-Tau immunofluorescence staining significantly increased in the cerebral cortex and hippocampus of mice experiencing *Pg*-induced periodontal disease. Notably, GV1001 demonstrated a substantial reduction in the staining intensity, as depicted in Figure 15.

### 2.19. GV1001 Significantly Inhibited the Expression of Pg Gingipains

**Figure 16:**
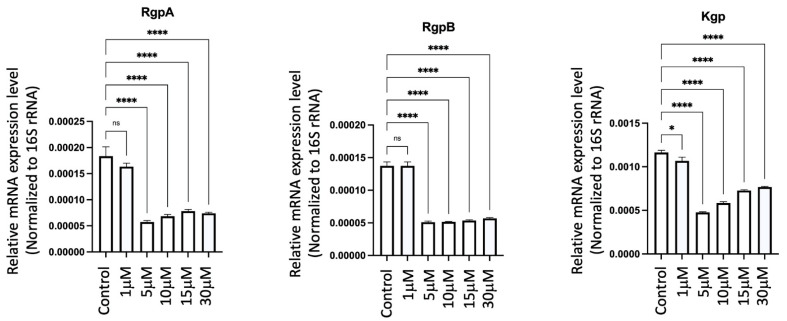
Effect of GV1001 on the expression of *Pg* gingipains RgpA, RgpB and Kgp in bacterial culture. *Pg* 16S rRNA served as the loading control. Statistical analysis was conducted through one-way analysis of variance (ANOVA), where ns indicates not significant, * indicates *p* < 0.05 and **** denotes *p* < 0.0001. The presented results represent the means ± SEM from three samples.

To elucidate the mechanisms behind the GV1001-induced reduction of Pg colony formation, Pg LPS, and gingipains in gingival tissues, arterial walls, and the brain, we examined the effect of GV1001 on the expression of Pg gingipains, which are essential bacterial proteases for tissue penetration and bacterial growth. Notably, GV1001 significantly inhibited the expression of gingipains in vitro, as demonstrated in Figure 16. At a low dose of GV1001 (5–30 μM), the expression of RgpA and RgpB was reduced five-fold compared to the control, while Kgp expression was reduced three-fold.

## 3. Discussion

In this study, we evaluated the effect of GV1001 on the development of atherosclerosis and AD-like conditions linked to *Pg*-induced periodontal disease in *ApoE*-deficient mice, and we found that GV1001 protected against *Pg*-induced periodontal disease and associated atherosclerosis and Alzheimer’s disease (AD)-like conditions. To the best of our knowledge, this study is the first report demonstrating a protective effect of GV1001 in both periodontal disease and atherosclerosis and its involvement in AD.

GV1001 exhibited an inhibitory effect on the development of Pg-induced periodontal disease by effectively inhibiting gingival inflammation and alveolar bone loss. Pg DNA aggregates and the formation of aggregates involving Pg LPS and gingipains in the gingival tissue are known to play a crucial role in inducing an inflammatory environment by triggering proteolysis, apoptosis, and the production of various inflammatory factors from gingival cells [46,47]. Indeed, GV1001 in bacterial culture reduced the expression of Pg gingipains RgpA, RgpB and Kgp, even at low concentrations of 5 μM of GV1001 (Figure 16), suggesting that GV1001 has a direct role in suppressing bacterial formation and colonization. However, we would like to clarify that our primary objective in this investigation was not to propose GV1001 as a direct treatment for periodontitis. Instead, our focus was on exploring whether GV1001 could prevent the development of atherosclerosis and AD-like conditions in mice with Pg-induced periodontal disease. 

Besides reducing inflammation and bacterial colony formation in the gingival tissue, GV1001 also inhibited osteoclastogenesis (Supplemental Figure S6). While the detailed mechanisms underlying the inhibition of osteoclastogenesis remain to be fully elucidated, it is hypothesized that GV1001’s anti-inflammatory properties may play a significant role in this process. Further research is warranted to unravel the specific pathways and molecular interactions involved in GV1001’s modulation of osteoclastogenesis and its subsequent impact on alveolar bone loss.

It is worth noting that although repeated topical application of *Pg* into the gingival pocket leads to the detection of *Pg* DNA in the mouse gingival pocket, systemic GV1001 administration did not alter the intensity of electrophoretic images of *Pg* DNA when compared to the control (Figure 1). Therefore, the inhibited number of *Pg* DNA aggregates in the gingival tissue caused by GV1001 is somewhat puzzling. However, given that the invasion of periodontal pathogens into gingival tissue and their growth are crucial events in the initiation and progression of periodontal disease [48], it is possible that GV1001’s inhibition of the expression of gingipains is associated with penetration of GV1001 through gingival barriers. The mechanisms of GV1001-induced inhibition of gingipain expression in *Pg* needs further investigation.

Atherosclerosis, characterized by plaque formation in the vascular intima, involves the excessive deposition of various components, including lipids, leukocytes, foam cells, mesenchymal cells, and vascular smooth muscle cells [49]. Our previous studies have indicated that ligature-induced periodontal disease can exacerbate atherosclerosis through systemic and vascular inflammation, even in the absence of significant changes in serum cholesterol levels [22,24]. Similarly, in line with other findings demonstrating atherosclerosis development through prolonged topical application of *Pg* in the oral cavity [50], our study revealed that *Pg*-induced periodontal disease notably increased the lipid deposition in the arterial wall of *ApoE*-deficient mice. *Pg*-induced periodontal disease led to the infiltration of *Pg, Pg* LPS, and gingipains in the arterial wall, concurrently with the induction of vascular inflammation and an increase in CD47 expression. GV1001 demonstrated a remarkable ability to suppress lipid deposition in the arterial wall, potentially by inhibiting the accumulation of *Pg* and *Pg* toxins. This effect is attributed, at least partly, to the mitigation of vascular inflammation. 

Additionally, GV1001 blocked EndMT and CD47 expression induced by TNF-α, a major proinflammatory cytokine present in both serum and the arterial wall, and *Pg* LPS in vitro. In macrophages, the promoter activity of CD47 was remarkably enhanced by TNF-α or *Pg* LPS, most likely via the activation of the NFκB pathway [30], but inhibited by GV1001 (Figure 11). These data suggest that there is a clear mitigating effect of GV1001 on atherosclerosis indirectly via suppressing local and systemic inflammation in *Pg*-induced periodontal disease. This is supported by the notable inhibition of atherogenesis in mice achieved through the administration of CD47-blocking antibodies, as reported by Kojima et al. [51]. 

It is well-established that chronic inflammation tends to decrease high-density lipoprotein (HDL) levels while elevating low-density lipoprotein (LDL) levels in the serum [52,53]. Interestingly, GV1001 demonstrated a significant reduction in total cholesterol (TC) and LDL levels, which could be associated with the observed inhibition of atherogenesis in mice with *Pg*-induced periodontal disease. The precise reasons for these alterations remain unclear, and further studies are warranted to gain a deeper understanding of the underlying mechanisms.

The blood–brain barrier (BBB) serves as a protective barrier that restricts the entry of substances, including drugs, from the bloodstream into the brain [54]. It has been suggested that *Pg* gingipains may enhance the permeability of microvascular endothelial cells [55], potentially leading to the penetration of *Pg* and the formation of *Pg* DNA aggregates in the brains of mice with *Pg*-induced periodontal disease. Our data indicate a higher aggregate number of *Pg* DNA and Kgp and RgpB in the cerebral cortex and hippocampus of mice with *Pg*-induced periodontal disease, while systemic administration of GV1001 significantly reduced the number of aggregates of *Pg* DNA, Kgp, and RgpB. The protective mechanism of GV1001 may be related to the protective BBB barrier function against *Pg* penetration due to its inhibitory effect on gingipain expression, or to suppressing the accumulation of *Pg* DNA and gingipains in mouse brains. These findings highlight the potential of GV1001 in addressing challenges associated with crossing the BBB in the context of AD.

Furthermore, in the current studies, a diverse set of experiments indicated that GV1001 administration appears to suppress neuroinflammation, inhibit the penetration of *Pg* into the brain, alleviate the accumulation of *Pg* LPS and gingipain aggregates, and reduce the levels of Aβ42 and p-Tau in the brains of mice with *Pg*-induced periodontal disease. AD patients often experience neuroinflammation, including alterations in cytokine profiles in the brain [44]. *Pg* has been identified as a risk factor for the accumulation of AD and dementia biomarkers, such as Aβ42 and p-Tau aggregates, in the human brain [45]. Previous reports suggest a close association between the development of human AD and the presence of *Pg* LPS and gingipains in the brain [35]. Given that drugs targeting a single molecular objective may face challenges in demonstrating beneficial effects in AD clinical trials [56,57], compounds simultaneously targeting multiple processes might offer paramount solutions against AD. Numerous studies, including ours, confirm that GV1001 stands out as a compound with the ability to simultaneously affect multiple cellular targets [11,12,58]. Overall, GV1001 is proposed as a potential solution against AD due to its ability to simultaneously target multiple processes, in contrast to drugs focusing on a single molecular target.

However, this study has several limitations. First, although *Pg* is a significant virulent factor in periodontal diseases, other bacteria such as *T. denticola* and *T. forsythia*, along with various other commensal bacteria, also contribute to periodontal diseases. Therefore, it is important to examine the roles of these other bacteria and their interactions with Pg. Second, it remains unclear whether the pocket space created by placing the ligature for one week is sufficient for *Pg* colonization. Moreover, we did not measure the pocket depth before the ligature placement. Although our study detected *Pg* DNA in the gingival pockets with PCR analysis and assumed they were *Pg* colonies, it was amplified Pg DNA, not live bacterial colonies, in the pocket. Further, the pocket volume for humans ranges from 0.0005 to 0.001 mL [59], which warrants further evaluation to determine the appropriate pocket volume in mice. Third, *Pg* is acid-resistant. Thus, the potential for *Pg* ingestion leading to bacteremia cannot be ruled out and requires further investigation. Moreover, this study did not include control groups such as PBS containing inactivated *Pg* or gingipains, which should be addressed in future studies.

## 4. Materials and Methods

### 4.1. Overall Experimental Design and Its Rationale for Evaluating the Preventive Effect of GV1001 on the Development of Pg-Induced Periodontal Disease in ApoE-Deficient Mice

Many studies have employed murine models to examine the effects of Pg on both local and systemic changes in various tissues [60,61]. A widely used method involves the topical application of live bacteria in the oral cavity, which helps assess *Pg’*s role in the onset of *Pg*-related systemic disorders [62]. However, this approach generally does not lead to significant periodontal disease [63,64]. Another technique involves injecting *Pg* lipopolysaccharide (LPS) into gingival tissues to induce periodontitis, though this method does not fully replicate Pg’s actual effects [65,66]. Additionally, placing Pg-coated ligatures in the gingival sulcus of mice is another model used to induce periodontitis [67,68], but the mechanical trauma from the ligatures can obscure the bacterial effects. Consequently, these animal models fail to accurately mimic human periodontitis, where *Pg* colonizes in the periodontal pockets. In our previous study, we created a mouse model where a small amount of *Pg* was topically applied directly into the gingival pocket, facilitating bacterial colonization [24]. This model was utilized to investigate the effects of GV1001 on both local and systemic disorders, including atherosclerosis and Alzheimer’s disease (AD)-like conditions, in *ApoE*-deficient mice.

### 4.2. Animals and Animal Welfare

To examine the impact of GV1001 on the progression of *Pg*-induced periodontal disease, we employed our previously established periodontitis model in *ApoE*-deficient mice. As depicted in Supplemental Figure S1, we acquired forty male *ApoE-*deficient mice, four weeks old, with a C57BL/6 genetic background, from Jackson Laboratory in Bar Harbor, ME. As wild-type mice do not develop atherosclerosis due to lack of low-density lipoprotein (LDL), we used *ApoE*-deficient mice in this study [22]. All mice were housed in a pathogen-free animal experimental facility at the University of California, Los Angeles, maintained under a 12 h light/dark cycle. They were fed a high-fat diet (HFD) from Research Diets, New Brunswick, NJ, USA, with free access to drinking water and food. Throughout the 11-week experiment, the health and behavior of the mice were monitored three times a week. During the ligature placement around the maxillary second molars and the topical application of either phosphate-buffered saline (PBS, pH 7.4) or Pg inoculation in the gingival pocket, a mixture of ketamine (100 mg/kg) and xylazine (5 mg/kg) was used as a general anesthetic. Carprofen (3 mg/kg) was administered post-ligature placement to minimize pain. The ketamine/xylazine mixture was given via intraperitoneal (i.p.) injection, and isoflurane was provided via inhalation.

To minimize suffering, all mice were administered ketamine/xylazine before euthanasia. The mice were euthanized via cardiac perfusion, and we monitored the heartbeat for 5 min to confirm death. All procedures were conducted in compliance with the institution’s policy and applicable regulations of the United States Department of Agriculture (USDA) Animal Welfare Act and the Public Health Service (PHS) policy. The Animal Research Committee (ARC) of the University of California, Los Angeles (UCLA) approved the experimental protocols under ARC Number 2019-057.

### 4.3. Creation of Gingival Pocket to Retain Topically Applied PBS or Bacteria in the Pocket and Groups of Animals

After a one-week quarantine, a 6–0 silk ligature was placed around the maxillary second molars (M2) under ketamine/xylazine anesthesia (100 and 5 mg/kg) for one week to create a gingival pocket for holding PBS or *Pg* (strain: W83). Post-ligature placement, all mice received 2 mg ampicillin and 2 mg neomycin daily by gavage for four days to achieve possible germ-free conditions and enhance *Pg* colonization. One week later, the ligature was removed (Figure 1), and the mice were divided into four groups as follows:

Group 1 (*n* = 10): Topical PBS application into the gingival pocket thrice per week for five weeks with the subcutaneous (sc) injection of PBS thrice per week for ten weeks (PBS + PBS); 

Group 2 (*n* = 10): PBS application into the gingival pocket thrice per week for five weeks with sc injection of GV1001 thrice per week for ten weeks (PBS + GV1001); 

Group 3 (*n* = 10): *Pg* application into the gingival pocket thrice per week for five weeks with the sc injection of PBS thrice per week for ten weeks (*Pg* + PBS); 

Group 4 (*n* = 10): *Pg* application into the gingival pocket thrice per week for five weeks with the sc injection of GV1001 thrice per week for ten weeks (*Pg* + GV1001).

Subcutaneous injections of PBS or GV1001 (2 mg/kg, sourced from GemVax/Kael, Inc., Sungnam-si, Republic of Korea) were administered one week before ligature placement in a volume of 0.1 mL (Supplemental Figure S1).

At the end of the experiment, the mice were sacrificed to assess the magnitude of periodontal disease development through (a) histological examinations (H&E staining of the periodontium), (b) gingival inflammation status, (c) expression levels of proinflammatory cytokines in gingival tissue, (d) presence of *Pg* DNA aggregates, *Pg* lipopolysaccharide (LPS), and gingipains in gingival tissue, and (e) alveolar bone loss using micro-computed tomography (μCT) analysis, along with systemic alterations as described in subsequent sections.

### 4.4. Preparation of PBS Containing 1% Methyl Cellulose, Culture of Pg and Topical Inoculation of PBS or Pg Directly into the Gingival Pocket, and the sc Injection of PBS or GV1001

To prepare 1% methyl cellulose in PBS, we added 1 g of methyl cellulose powder to 33 mL of heated PBS (80 °C). The mixture was agitated until the methyl cellulose was thoroughly wetted and evenly dispersed in PBS. For complete solubilization, an additional 67 mL of PBS was added to prepare the PBS containing 1% methyl cellulose. The solution was then cooled on ice for 40 min until it reached a temperature of 0–5 °C. Subsequently, the solution was vigorously agitated again for 30 min and kept at 4 °C until use for the preparation of the Pg suspension and control PBS. 

The *Pg* strain W83 (obtained from Dr. Gena D. Tribble at University of Texas Health Science Center, Houston, TX) was grown in tryptic soy broth (TSB) supplemented with 5 μg/mL of hemin, 0.5 μg/mL vitamin K1, and 0.05% L-cysteine in an anaerobic chamber at 37 °C until OD600 of ~1.5 [~7.3 × 10^9^ colony forming units/mL (CFU/mL)]. *Pg* culture was pelleted at 12,500 rpm for 10 min at 15 °C in a JA-25.5 rotor by Beckman Avanti J-E centrifuge (Brea, CA) and washed once with PBS without adding additional reducing agent before the bacterial pellet was suspended in a sterile 1% methyl cellulose solution to yield a final 5 × 10^11^ CFU/mL concentration. The aforementioned procedures were performed outside the anaerobic chamber. Fresh *Pg* culture preparations were conducted thrice a week for five consecutive weeks. Ten µL PBS prepared in methyl cellulose solution or 10 µL methyl cellulose solution containing 5 × 10^9^ CFU *Pg* was topically applied directly into the gingival pocket using the microvolume micropipette three times per week for five weeks under a microscope. PBS or GV1001 was administered thrice per week via sc injection as shown in Supplemental Figure S1. We topically applied *Pg* suspension into the pocket three times for 5 weeks to establish the *Pg* colony formation (or detect the *Pg* DNA) in the gingival pocket at 4 weeks after the final Pg application, as we found it was necessary in our pilot study.

### 4.5. Sample and Tissue Collections

Whole blood, gingival swab samples, gingival tissue, aortic roots, heart, brain, entire arteries, and maxillae were collected from the mice and processed or stored as described previously [11]. 

### 4.6. Frozen Sectioning

The heart samples were frozen sectioned for immunofluorescence staining of the aortic root region as described previously [11].

### 4.7. Detection of Pg DNA Aggregates from the Gingival Pocket

Six randomly chosen swab samples from each group were stored in 200 µL PBS. Genomic DNA was extracted using the Qiagen QIAamp DNA Micro kit (Qiagen, Inc., Hilden, Germany) following treatment with proteinase K (Thermo Fisher Scientific, Inc., Waltham, MA, USA). The purified, protein-free DNA was dissolved in 20 µL distilled water. A 5 µL aliquot of this DNA was diluted 10-fold, and the diluted sample was boiled for 5 min. The resulting lysate (4 µL) was used as a template for PCR, with the DNA amount representing 1/50th of the original swab sample. PCR reactions were carried out as previously described [24,69] using primers specific for Pg W83 16S rDNA. Each reaction included an initial denaturation at 95 °C for 8 min, followed by 50 cycles of 95 °C for 30 s, 56 °C for 30 s, and 72 °C for 40 s. PCR was performed using the SimpliAmp Thermal Cycler (Thermo Fisher Scientific, Inc., Waltham, MA, USA). Amplified products were detected by electrophoresis on a 1% agarose gel containing ethidium bromide, run at 110 V for 20 min. A 1 Kb plus DNA ladder (Thermo Fisher Scientific, Waltham, MA, USA) was used as a marker. Purified Pg DNA (1000 CFU) served as a positive control to quantify the CFU of Pg from each swab, while PCR without template DNA served as a negative control. The density of the amplified DNA was analyzed using ImageJ software Version 1.52 (National Institutes of Health).

### 4.8. Micro-Computed Tomography (μCT) and Histological Analysis of Maxillae

The maxillae, once securely positioned, underwent microCT scanning (Skyscan1275, Bruker-microCT Systems, Allentown, PA, USA) with a voxel dimension of 20 μm^3^ and a 0.5 mm aluminum filter. Utilizing NRecon software (Version 2.0) and CTVol software (Version 2.0) (Bruker-microCT Systems, Billerica, MA, USA), two-dimensional slices from each maxilla were amalgamated to generate a three-dimensional reconstruction. The area of interest was delineated by scrutinizing orthogonal projections of each slice through the Dataviewer software (Version 1.5.1) (Bruker-microCT Systems, Billerica, MA, USA). The extent of bone resorption, measured as the distance from the palatal and mid-buccal cement–enamel junction (CEJ) to the alveolar bone crest (ABC) of the second molars, was assessed by two experts using the CTAn software (Version 1.20.8) (Bruker-microCT Systems, Billerica, MA, USA). Another individual validated the readings in a blinded manner.

### 4.9. Histological and Immunofluorescence Analysis

Following μCT scanning, the maxillae were decalcified, embedded in paraffin, and sectioned, with staining with hematoxylin and eosin as described previously [11]. For tartrate-resistant acid phosphatase (TRAP) staining, maxillary sections were stained using a solution containing Naphthol AS-TR phosphate sodium salt (Sigma-Aldrich, St. Louis, MO, USA) and Fast Violet Red dye (Sigma-Aldrich) in buffer, followed by counterstaining with hematoxylin. Digital images of the stained sections were captured using the DP72 microscope (Olympus Corporation, Tokyo, Japan). Clinical attachment loss (CAL) was assessed under the microscope by measuring the distance from the CEJ to the base of the pocket depth. The readings were validated in a blinded manner by another individual.

For immunofluorescent staining, the paraffin sections of maxillae and brain, along with frozen sections of aortic roots, were treated with primary antibodies, including TNF-α (Abcam, ab6671, Cambridge, UK), IL-6 (Thermo Fisher Scientific, Waltham, MA, USA), IL-1β (Thermo Fisher Scientific, Waltham, MA, USA), Pg LPS (CD Creative Diagnostics, Shirley, NY, USA), Pg gingipains (rabbit polyclonal antibody prepared by our laboratory), CD47 (Cluster of Differentiation 47, Rosemont, IL, USA), αSMA (alpha smooth muscle actin, Abcam, Cambridge, UK), Aβ42 (rabbit polyclonal antibody, Thermo Fisher Scientific, Waltham, MA, USA), p-Tau (mouse monoclonal antibody, Thermo Fisher Scientific, Waltham, MA, USA), or MAP-2 (mouse polyclonal antibody, Thermo Fisher Scientific, Waltham, MA, USA). Subsequently, fluorometric detection was carried out using Alexa Fluor 594-conjugated secondary antibodies (Thermo Fisher Scientific, Waltham, MA, USA). The sections were then mounted on slides with VECTASHIELD TM anti-fade mounting medium containing 4′,6-diamidino-2-phenylindole (DAPI) (Vector Laboratories, H1200, Burlingame, CA, USA). Immunofluorescent images were captured using a confocal fluorescent microscope (Carl Zeiss, LSM 700, Oberkochen, Germany).

### 4.10. Osteoclastogenesis Assay

The influence of GV1001 on osteoclast formation was evaluated using mouse macrophage RAW 264.7 cells obtained from ATCC (Manassas, VA, USA). These cells were cultured in Dulbecco’s Modified Eagle Medium (DMEM; Thermo Fisher Scientific, Waltham, MA, USA) supplemented with 10% fetal bovine serum (FBS; Thermo Fisher Scientific, Waltham, MA, USA). Osteoclastogenesis was induced by treating the cells with RANKL (R&D Systems, Allendale, NJ, USA), either alone or in conjunction with GV1001 (at concentrations of 0, 1, 5, or 10 μg/mL), for a duration of five days. Subsequently, the cells were fixed using ice-cold 70% ethanol for 15 min, followed by staining for tartrate-resistant acid phosphatase (TRAP), a hallmark indicator of osteoclasts, for five minutes, as per the manufacturer’s instructions provided with the TRAP staining kit (Sigma-Aldrich, St. Louis, MO, USA).

### 4.11. Antibody Production for Pg Kgp and RgpB

Due to the limited availability of anti-*Pg* (strain W83) gingipain antibodies, polyclonal antibodies against Pg gingipains (Kgp and RgpB) were generated following previously described methods [24]. These newly produced antibodies were utilized to detect gingipains in the gingival tissue, arterial wall, and brain.

### 4.12. Serum Lipid and Proinflammatory Cytokines Measurement in Mouse Serum

Levels of total cholesterol, triglycerides, high-density lipoprotein (HDL), and non-HDL were measured using enzymatic assay kits in the UCLA Cardiovascular Core Facility. The levels of proinflammatory cytokines were detected, as reported previously [22,30]. 

### 4.13. Quantitative Real-Time Polymerase Chain Reaction

Total RNA was extracted from mouse gingival, aortic, and brain tissues using the RNeasy micro kit (Qiagen, Inc., Hilden, Germany) and reverse transcribed as described previously [11].

### 4.14. Fluorescence In Situ Hybridization (FISH) Analysis for the Detection of Pg DNA Aggregates in the Gingival, Aortic Wall, and Brain Tissue

In situ hybridization was performed on frozen heart sections and formalin-fixed and paraffin-embedded gingival and brain tissue sections using Alexa Fluor 488 (Thermo Fisher Scientific, Waltham, MA, USA) labeled oligonucleotide probes specific for *Pg* (5′-CAATACTCGTATCGCCCGTTATTC-3′). FISH was then performed as reported previously [70,71,72]. Stained slides were dried overnight and viewed using the Fluoview FV200i confocal fluorescent microscope (Olympus Corporation, Tokyo, Japan). 

### 4.15. En Face Analysis

The en face analysis for determining the amount of lipid deposition in arterial wall was conducted as described previously [73]. 

### 4.16. Cell Culture and Reagents

Human umbilical vein endothelial cells (HUVEC; Lonza, Switzerland) and human coronary artery smooth muscle cells (HCASMCs; Lonza, Switzerland) were cultured in endothelial basal medium-2 and smooth muscle cell growth basal medium (Lonza, Switzerland), respectively. RAW 264.7 cells were cultured in Dulbecco’s Modified Eagle Medium (DMEM, ATCC, Manassas, VA, USA) supplemented with 10% fetal bovine serum (FBS). The medium was renewed every 48 hours. THP-1 cells (ATCC, Manassas, VA, USA) were cultured in RPMI 1640 medium (Sigma-Aldrich, St. Luis, MO, USA) supplemented with 10% FBS. Cells were cultured at 37 °C and in a 5% (*v*/*v*) CO_2_ of air atmosphere with humidity.

### 4.17. Induction of EndMT

The EndMT was performed as described previously [11].

### 4.18. Western Blotting

The Western blotting was performed as described previously [11]. 

### 4.19. CD47 Promoter Activity Assay

After construction of CD47-luciferase vector, HCASMCs were transfected with 45 ng of the CD47-luciferase vector and 5 ng of the reference vector using Lipofectamine 2000 (Invitrogen, Waltham, MA, USA) for 48 h. The cell lysates were collected, and dual luciferase activity was measured with the Dual-Luciferase Reporter Assay System (Promega, Madison, WI, USA), according to the manufacturer’s instructions. Relative luciferase activity (Firefly/Renilla luciferase ratio) was quantified as the CD47 promoter reporter activity as described previously [23]. 

### 4.20. Oil O Red Staining

THP-1 cells were differentiated into macrophages by incubation with 200 nM phorbol 12-myristate 13-acetate (PMA, Sigma-Aladrich, St. Louis, VA, USA) in chamber slides (Thermo Fischer Scientific, Waltham, MA, USA) for 24 h. The differentiated THP-1 cells were then exposed to GV1001 (10 μg/ml), TNF-α (100 ng/ml), *Pg* LPS (10 μg/ml), TNF-α (100 ng/ml) plus GV1001 (10 μg/ml), *Pg* LPS (10 μg/ml) plus GV1001 (10 μg/ml) for 24 h. After the exposure, the cells were treated with dil-ox-LDL for 6 hours and fixed with 4% paraformaldehyde in PBS for 15 min [74].

### 4.21. Determination of the Effect of GV1001 on the Gingipain Expression in Pg

The early log phase cultures of *Pg* at O.D._600_ = 0.1 in the tryptic soy broth (BD Biosciences, Franklin Lakes, CA, USA) containing 100-fold-diluted Vitamin K1 Hemin solution (BD Biosciences, Franklin Lakes, NJ, USA) and 0.05% cysteine were incubated with 0, 1, 5, 10, 15, and 30 µM of GV1001 for 24 h. in the anaerobic chamber at 37 °C. One ml of bacterial culture from each GV1001 treatment was extracted for RNA using the NucleoSpin RNA purification kit (Macherey-Nagel, Duren, Germany) according to the manufacturer’s instruction. The RNA from the NucleoSpin kit was subjected to additional DNase I digestion (RNase free, Thermo Fisher Scientific, Waltham, MA, USA) for 30 min at 37 °C before extraction with phenol/chloroform at pH 4.3 to remove residual chromosomal DNA. The resulting RNA solution was precipitated by 2.5 volumes of ethanol in the presence of 0.1 volume of 3 M sodium acetate at −80 °C for 30 min before being centrifuged at 4 °C for 20 min. The RNA pellets were washed with 70% ethanol once, then centrifuged at 4 °C for 20 min before drying. A total of 1 µg of RNA from each sample was used for cDNA synthesis via the iScript Supermix (Bio-Rad Laboratories, Hercules, CA, USA) according to the manufacturer’s suggested program (25 °C for 5 min, 46 °C for 40 min, and 95 °C for 1 min) in the presence and absence of reverse transcriptase to verify no contamination by chromosomal DNA. The diluted cDNA samples were used for RT-qPCR with specific primer sets (Table S1 in Supplemental Materials) for the detection of *RgpA*, *RgpB*, and *Kgp* gene expression using PowerUp™ SYBR-Green Master Mix (Thermo Fisher Scientific, Waltham, MA, USA) or TaqMan primers (Applied Biosystems; Thermo Fisher Scientific, Waltham, MA, USA) according to the manufacturers’ protocols. The sequences of the primers used for RT-qPCR are presented in Table S1. *Pg* 16S ribosomal mRNA served as a control, and the fold induction was calculated using the comparative ΔCq method and presented as relative transcript levels (2^−ΔΔCq^).

### 4.22. Statistical Analyses

The graphs were generated utilizing GraphPad Prism software, and statistical analyses were conducted using GraphPad Prism 9 (GraphPad Software, Boston, MA, USA). For multiple comparisons, we employed one-way ANOVA with the Newman–Keuls test. A significance threshold of *p* < 0.05 was applied. All in vitro findings were validated through a minimum of three independent experiments. Error bars denote the mean  ±  standard error (SE). According to our power analysis, we required 10 mice per group to detect statistically significant differences between the control and experimental groups. The calculation was based on continuous variables assuming a symmetric distribution and α = 0.05 and β = 0.2 with a power at 80%.

## 5. Conclusions

The inoculation of *Pg* into the gingival pockets of the molars resulted in periodontal disease in *ApoE*-deficient mice, characterized by increased gingival pocket depths, local inflammation, alveolar bone loss, and systemic inflammation. Systemic administration of GV1001 significantly inhibited the development of *Pg*-induced periodontal disease and demonstrated the ability to inhibit vascular inflammation, most likely by preventing the accumulation of *Pg* DNA, lipopolysaccharides (LPSs), and gingipains in the arterial wall. This inhibition contributed to the prevention of atherosclerosis development in *ApoE*-deficient mice. Moreover, GV1001 was found to block lipid deposition associated with *Pg*-induced periodontal disease, potentially by reducing vascular inflammation, inhibiting the conversion of arterial endothelial cells to mesenchymal cells (EndMT), suppressing the expression of CD47 from arterial smooth muscle cells, and inhibiting foam cell formation. GV1001 also reduced the accumulation of biomarkers associated with Alzheimer’s disease (AD) in the brains of mice with *Pg*-induced periodontal disease, suggesting a potential preventive effect on AD in patients with periodontitis. However, it is important to note that further studies, including clinical trials, are necessary to validate the effects of GV1001 on systemic disorders associated with periodontitis.

## Data Availability

Data are available upon request to the corresponding author.

## Supplementary Materials

The supporting information can be downloaded at: https://www.mdpi.com/article/10.3390/ijms25116126/s1.
