# Supplementary material for: hTERT Peptide Fragment GV1001 Prevents the Development of Porphyromonas gingivalis-Induced Periodontal Disease and Systemic Disorders in ApoE-Deficient Mice
Source: Int J Mol Sci. 2024 Jun 1;25(11):6126. doi: 10.3390/ijms25116126 (PMC11172542; doi:10.3390/ijms25116126)
Supplement: Supplementary file 1 [file ijms-25-06126-s001.zip › ijms-2981453-supplementary.pdf]

Supplemental Materials

hTERT Peptide Fragment GV1001 Prevents the Development of *Porphyromonas gingivalis*-Induced Periodontal Disease and Systemic Disorders in *ApoE*-Deficient Mice

Wei Chen [1,†], Sharon Y. Kim [1,†], Alicia Lee [1], Yun-Jeong Kim [1,‡], Chungyu Chang [2], Hung Ton-That [2], Reuben Kim [1,3], Sangjae Kim [4] and No-Hee Park [1,4,5,*]

[1] The Shapiro Family Laboratory of Viral Oncology and Aging Research, UCLA School of Dentistry,
714 Tiverton Ave, Los Angeles, CA 90095, USA; chenwei304@ucla.edu (W.C.); skim@dentistry.ucla.edu (S.Y.K.); alicialee15@g.ucla.edu (A.L.); yjbest01@snu.ac.kr (Y.-J.K.); rkim@dentistry.ucla.edu (R.K.)

[2] Section of Oral Biology, UCLA School of Dentistry, 714 Tiverton Avenue, Los Angeles, CA 90095, USA; jchang@dentistry.ucla.edu (C.C.); htonthat@dentistry.ucla.edu (H.T.-T.)

[3] UCLA Jonsson Comprehensive Cancer Center, 10833 Le Conte Ave, Los Angeles, CA 90095, USA

[4] Teloid Inc., 920 Westholme Avenue, Los Angeles, CA 90024, USA; chiron99@hanmail.net

[5] Department of Medicine, David Geffen School of Medicine at UCLA, 10833 Le Conte Ave, Los Angeles, CA 90095, USA

* Correspondence: nhpark@g.ucla.edu

† These authors contributed equally to this study.

‡ Current address: Department of Periodontology, Gwanak Dental Hospital, School of Dentistry and Dental Research Institute, Seoul National University, Seoul 08826, Republic of Korea.

Supplemental Figures:

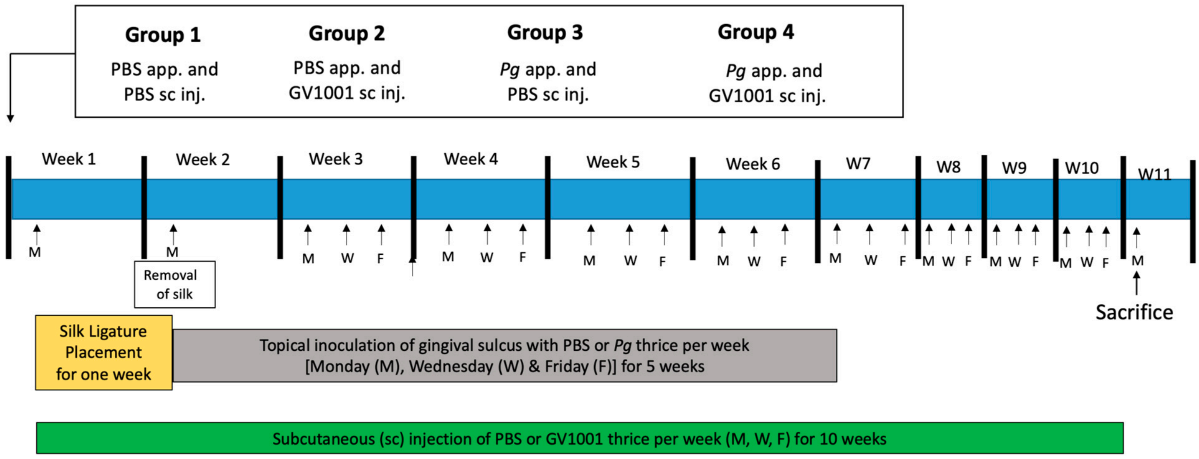

Supplemental Fig. S1. Experimental design for investigating the effect of systemic GV1001 on the development of *Pg*-induced periodontitis. After one week quarantine, we divided the mice into four groups: (1) Group 1: Inoculation of gingival pocket with PBS thrice per week for five weeks with sc injection of PBS thrice per week for ten weeks (PBS + PBS); (2) Group 2: Inoculation of gingival pocket with PBS thrice per week for five weeks with sc injection of GV1001 (2mg/kg) thrice per week for ten weeks (PBS + GV1001); Group 3: Inoculation of gingival pocket with *Pg* thrice per week for five weeks with sc injection of PBS thrice per week for ten weeks (*Pg* + PBS); (4) Group 4: Inoculation of gingival pocket with *Pg* thrice per week for five weeks with sc injection of GV1001 (2mg/kg) thrice per week for ten weeks (*Pg* + PBS).

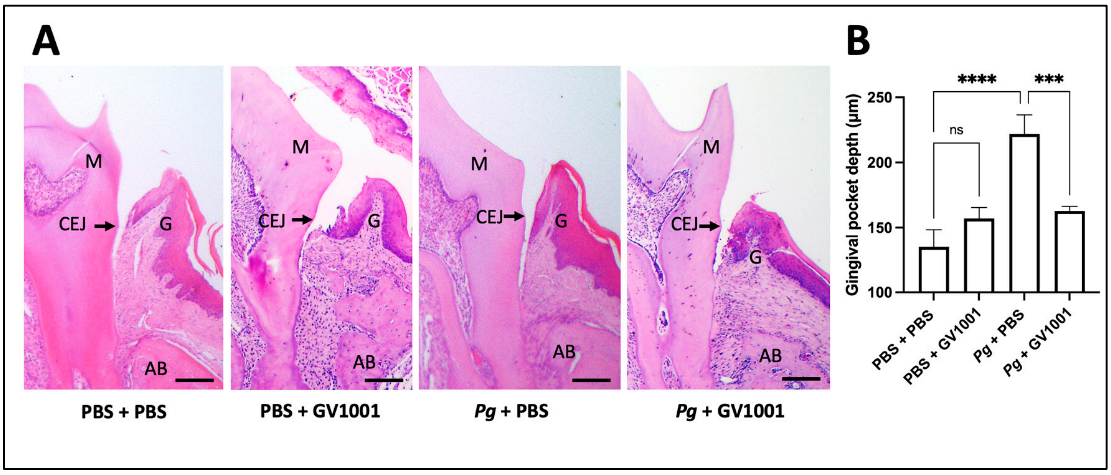

Supplemental Fig. S2. (**A**) Representative images of hematoxylin and eosin staining of the maxillary second molars and periodontal tissue. Black arrows indicate the cemento-enamel junction (CEJ). (**B**) Depth of gingival pocket histologically measured at the mesiobuccal pocket of the maxillary second molars from CEJ to the base of pocket using ImageJ. Results represent the means ± SEM. Statistical

analysis was performed with one-way ANOVA. Ns, not significant; ***P<0.001; ****P<0.0001. M: Molar; AB: Alveolar Bone; G: Gingiva. Scale bar: 100 μm

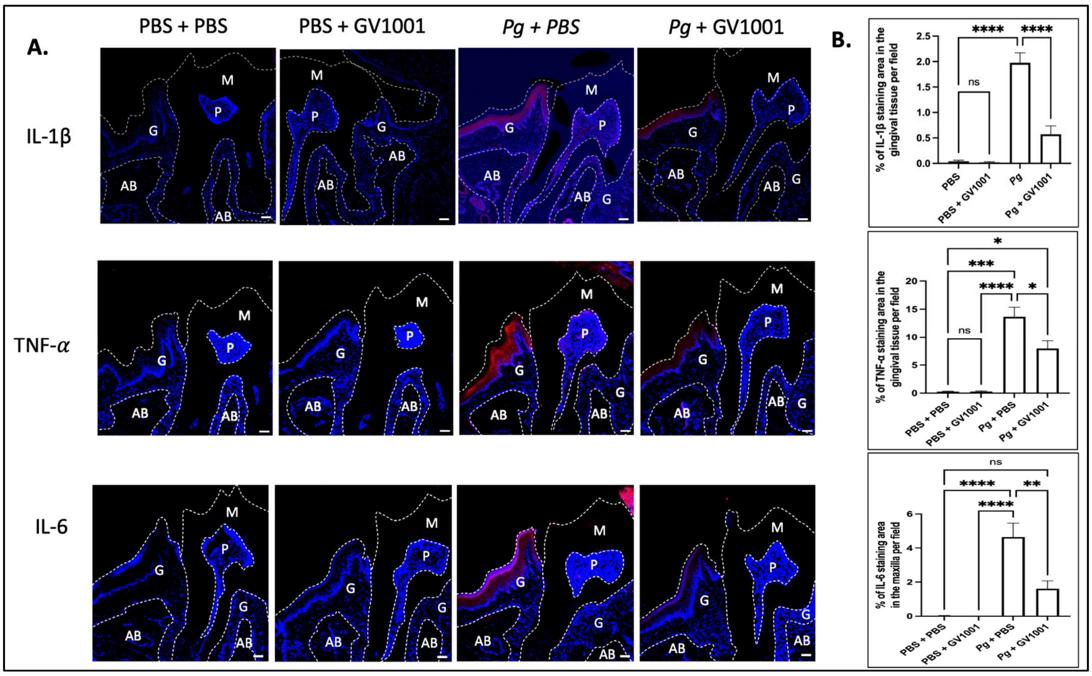

Supplemental Fig. S3. (A) Representative immunofluorescent staining images of proinflammatory cytokines (IL-1β, TNF-α, and IL-6) in gingival tissue. AB: Alveolar Bone; G: Gingival tissue; M: Molar; P: Pulp. The cytokines were stained in red color, and the blue dots are nuclei of cells stained with 4′,6-diamidino-2-phenylinodole (DAPI). The stained areas were analyzed with the ImageJ. (**B)** Results represent the means ± SEM. We performed the statistical analysis with one-way ANOVA. ns, not significantly different; * P<0.05; ** P<0.01; *** P<0.001; **** P<0.0001. Scale bar: 100 μm, M: Molar; AB: Alveolar Bone; G: Gingival tissue. Bar scale: 100μm

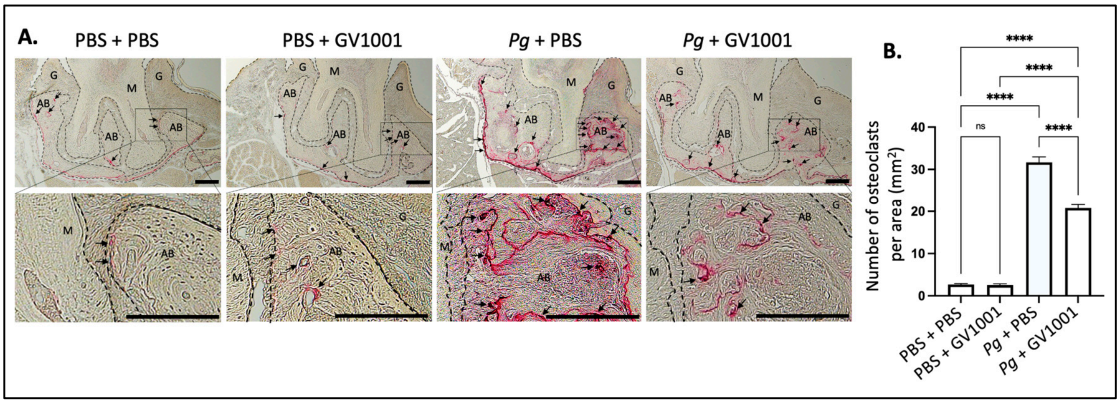

Supplemental Fig. S4. (**A**) Representative TRAP staining images of periodontium of mice receiving with or without topical *Pg* inoculation, along with PBS or GV1001 administration. Dark arrows indicate osteoclasts. (**B**) The number of osteoclasts per surface area (mm^2) and the results represent the means ± SEM performed in four samples. Statistical analysis was performed with one-way ANOVA. ns, not significantly different; ****, P<0.0001, Scale bar size: 200 μm

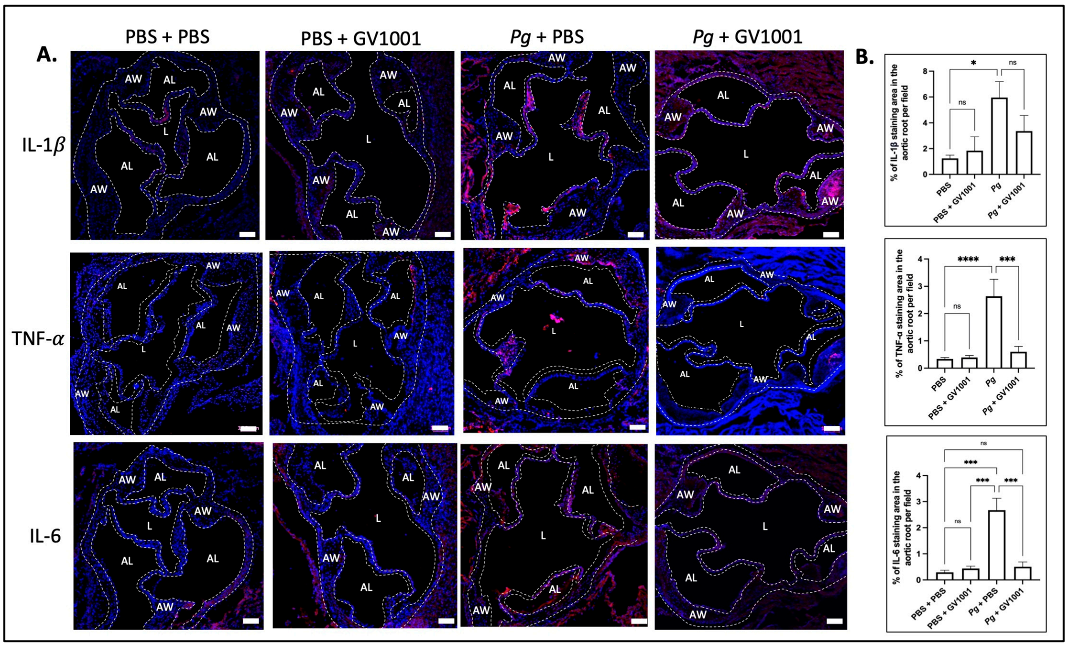

Supplemental Fig. S5. (**A**) Representative immunofluorescent staining images of the IL-1β, TNF-α, and IL-6 in the arterial wall of aortic roots of mice. The inflammatory cytokines were shown with bright red color, and the nuclei were counterstained with DAPI (blue dots). AW: Arterial Wall; L: Lumen; AL: Arterial Leaflet. (**B**) Results represent the means ± SEM of five samples in each group. Statistical analysis was performed with one-way ANOVA. ns, not significantly different; * P<0.05, *** P<0.001, **** P<0.0001. Scale bar: 100μm.

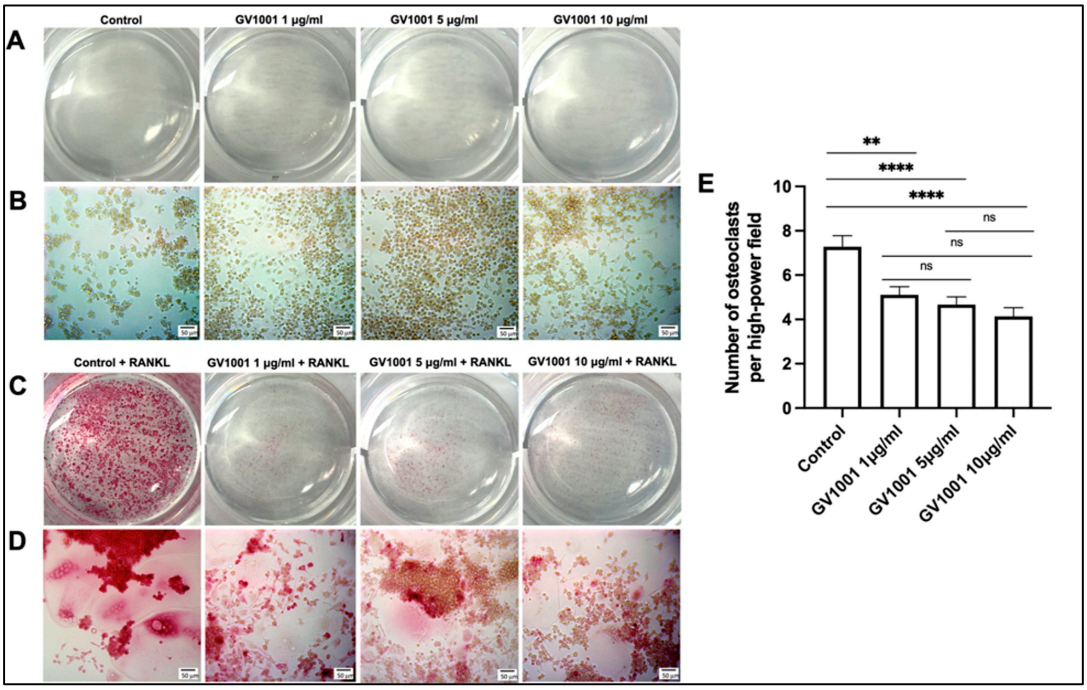

Supplemental Fig. S6. Effect of GV1001 RANKL-induced conversion of murine macrophages to osteoclasts *in vitro*. (**A**) Low-power images and (**B**) representative images of high-power fields of RAW 264.7 cells treated with 0, 1, 5, or 10 μg/ml of GV1001 without RANKL exposure. (**C**) Low-power images and (**D**) representative images of high-power fields of RAW 264.7 cells treated with 0, 1, 5, and 10 μg/ml of GV1001 with RANKL. The high-power field images of the stained sections were obtained using the DP72 microscope (Olympus Corporation). (**E**) Quantitative analysis showing the number of osteoclasts per high-power field averaged from 2 wells per group (18 high-power field images per well were averaged). Statistical analysis was performed with one-way ANOVA. Data are shown as mean ± SEM. **P<0.01 ****P<0.0001. Scale bar: 50 μm.

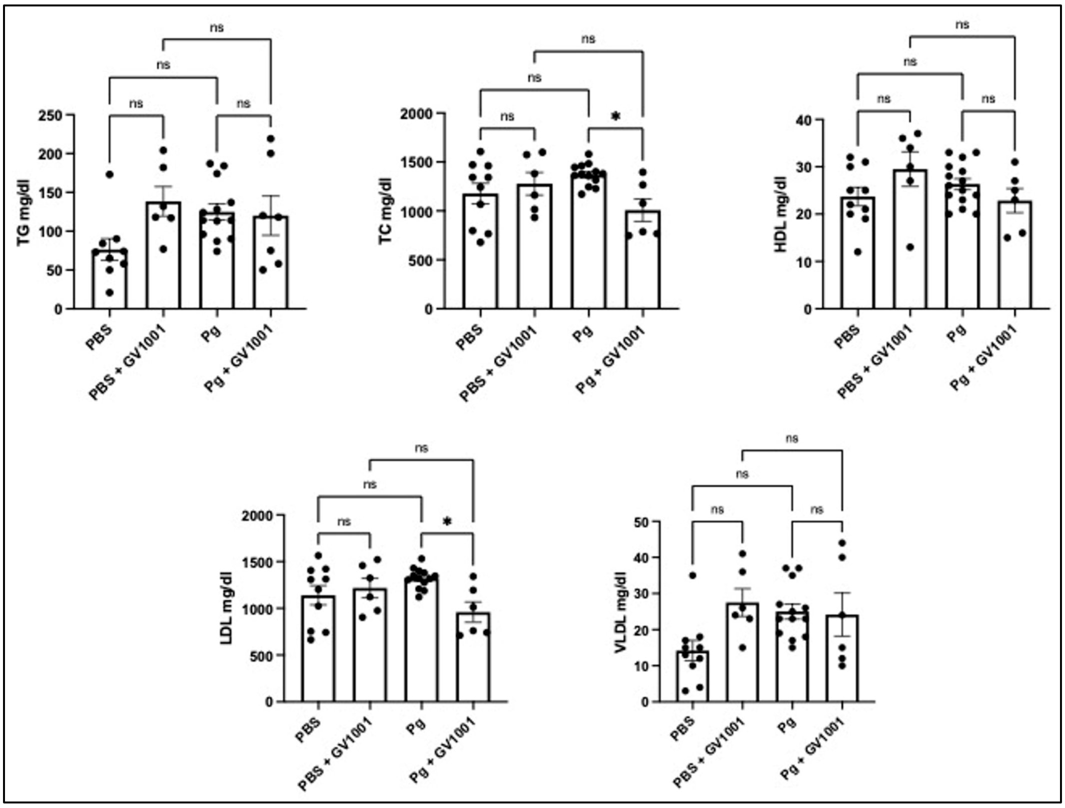

Supplemental Fig. S7. Alterations of serum cholesterol profiles by GV1001. Triglyceride (TG), HDL, and VLDL levels were not altered by systemic GV1001 in *Pg*-inoculated mice, but TC and LDL levels were significantly reduced in comparison to the mice with *Pg* inoculation with systemic PBS administration.

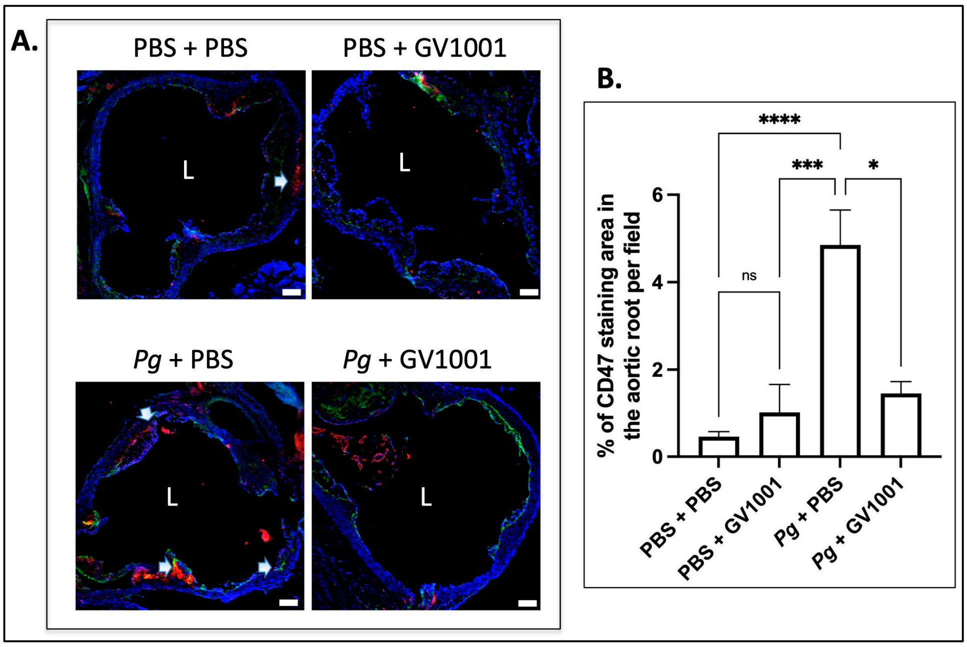

Supplemental Fig. S8. (**A**) Representative immunofluorescent staining images of CD47 in mouse aorta. A bright red color indicates CD47. Blue dots are nuclei stained with DAPI. AW: Aortic Wall; L: Lumen; α-SMA: the smooth muscle cell marker shown by green color. Scale bar: 100 µm. (**B)** Quantification of areas stained CD47 using ImageJ analysis. ns, not significantly different; * P < 0.05; *** P<0.001; **** P<0,0001. Scale bar: 25m.

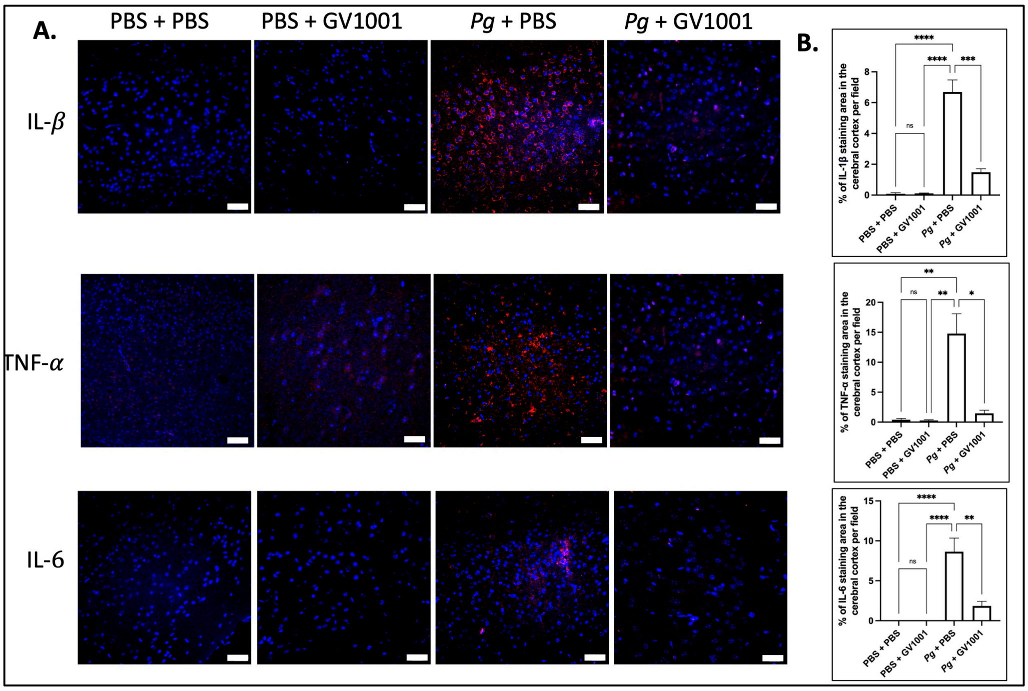

Supplemental Fig. S9. **(A)** Representative immunofluorescent staining images capturing the presence of IL-1β, TNF-α, and IL-6 in the cerebral cortex of the mouse brain. The proinflammatory cytokines are visualized in a vivid red color, while the nuclei are counterstained with DAPI, appearing as blue dots. **(B)** The results presented in this figure represent the means ± SEM of five samples within each group. Statistical analysis was conducted using one-way analysis of variance (ANOVA). The categories for significance are denoted as follows: ns, not significantly different, * P<0.05, ** P<0.01, *** P<0.001, **** P<0.0001. The scale bar in the images is 40 μm.

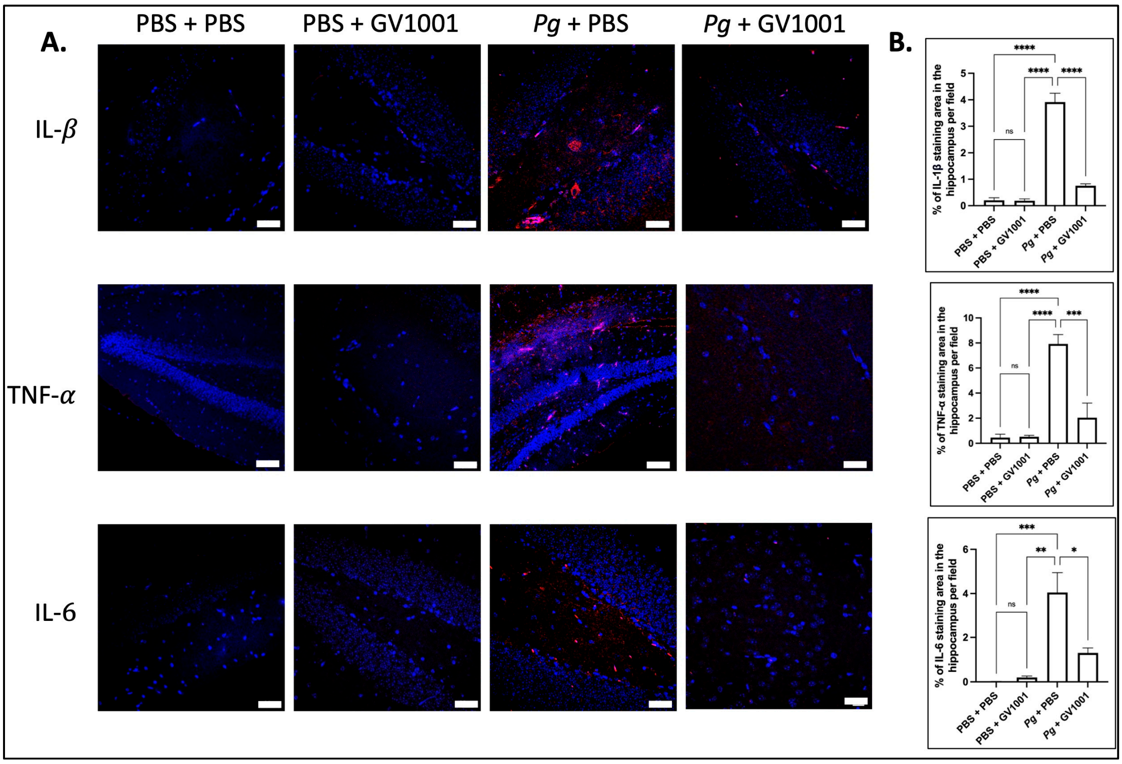

Supplemental Fig. S10. **(A)** Representative immunofluorescent staining images results for the inflammatory cytokines IL-1β, TNF-α, and IL-6 within the hippocampus of the mouse brain. The inflammatory cytokines are visualized in a vibrant red color, while the nuclei are counterstained with DAPI, appearing as blue dots. **(B)** The data presented in this figure represent the means ± SEM of five samples in each group. Statistical analysis was conducted using one-way analysis of variance (ANOVA). Results are categorized as follows: ns, not significantly different, * P<0.05, ** P<0.01, *** P<0.001, **** P<0.0001. The scale bar in the images is 40 μm.

Supplemental Table:

Table S1. Sequences of the primers for quantitative reverse transcription-polymerase chain reaction (RT-qPCR).

Genes	Forward primer 5′-3′	Reverse primer 5′-3′
mIL-1β	CACAGCAGCACATCAACAAG	GTGCTCATGTCCTCATCCTG
mTNF-α	TCAGGTTGCCTCTGTCTCAG	GCTCTGTGAGGAAGGCTGTG
mIL-6	TGGGACTGATGCTGGTGACA	GCCTCCGACTTGTGAAGTGGT
mGAPDH	AGCTTGTCATCAACGGGAAG	TTTGATGTTAGTGGGGTCTCG
hCD31	GCAACACAGTCCAGATAGTCGT	GACCTCAAACTGGGCATCAT
hFSP1	GCTCAACAAGTCAGAACTAAAGGAG	GCAGCTTCATCTGTCCTTTTC
hCD47	TATCCTCGCTGTGGTTGGACTG	TAGTCCAAGTAATTGTGCTAGAGC
hGAPDH	AGCCACATCGCTCAGACAC	GCCCAATACGACCAAATCC
RgpA	CGAAGCCAAGGTTGTGCTCG	AGGATCGGCATTGGCCGG
RgpB	GAGTATCGCTGATGAAACGAACTTGACG	CATATCGAAGATCGTCAGCCCG
Kgp	GGACTACTATTGGAGTGTGGGTGC	GAGCACCAATATGGGTAATATTGCCG
16S	CTGAGCGCTCAACGTTCAGCC	GTGGAAGCTTGACGGTATATCGCAA

Original gel and Western blot pictures

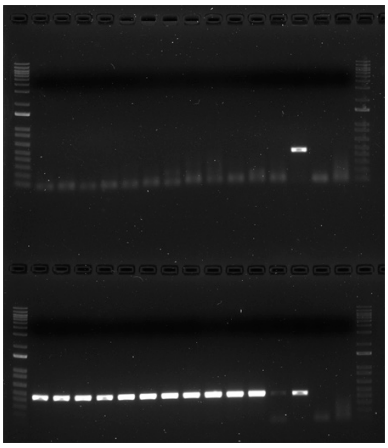

Original gel picture of Figure 1

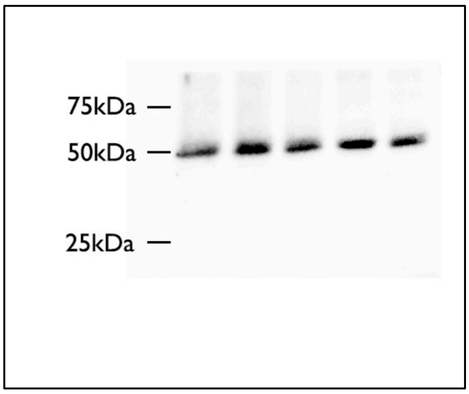

Original Western blotting picture of Figure 11C. CD47

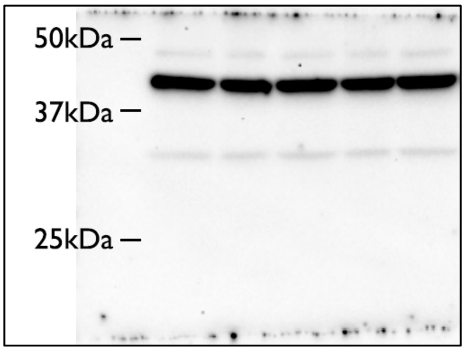

Original Western blotting pictures of Figure 11C. β-actin
